# Quantifying the Number of Dark, Gray, and Bright States as a Function of the Spectral Overlap in Polaritonic Systems

**DOI:** 10.1021/acs.nanolett.5c06424

**Published:** 2026-05-27

**Authors:** Rahul Bhuyan, Ilia Sokolovskii, Clara Schäfer, Gerrit Groenhof, Karl Börjesson

**Affiliations:** † Department of Chemistry and Molecular Biology, University of Gothenburg, Gothenburg 41390, Sweden; ‡ Nanoscience Center and Department of Chemistry, University of Jyväskylä, Jyväskylä 40014, Finland

**Keywords:** light−matter interaction, strong coupling, ideal polariton, exciton reservoir

## Abstract

Strong exciton–photon coupling can steer photophysical processes. However, the picture where it leads to two polaritonic states and a manifold of optically dark states is now questioned. Instead, a picture where inhomogeneous broadening results in a partial photonic contribution to the dark states has gained ground. To understand the consequence of these dark and so-called gray states, they first need to be experimentally quantified. Here we use angle-resolved emission, coupled to rate equation modeling, to quantify the absolute number of states in optical cavities. In addition, we also estimate the fraction of states that are gray by means of computer simulations. We find that the number of gray states is proportional to the overlap between the energy of the dark and polaritonic states. This knowledge can be applied to all previous and future studies to interpret how detuning affects the relative number of dark and gray states and, consequently, polaritonic dynamics.

## Graphical abstract

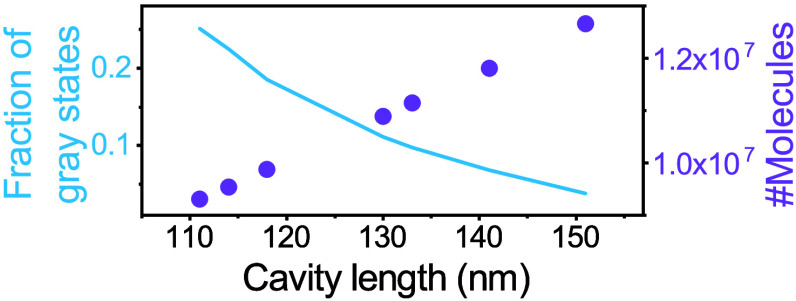

Strong coupling between excitons and photons leads to the formation of hybrid light-matter quasiparticles called polaritons. These hybrid states inherit properties from both constituents, enabling them to delocalize across all molecules coupled to the photon.^1–6^ This concept has attracted significant attention in recent years due to its broad applications, which include enhancing energy transfer,^7–12^ exciton diffusion,^13,14^ reverse intersystem crossing,^15,16^ singlet fission and triplet fusion,^17–20^ organic electronics,^21–27^ modifying chemical reactivity,^28–31^ and enabling room-temperature Bose–Einstein condensation.^32,33^ Despite these exciting advances, a fundamental understanding of strong light–matter interaction such as the nature of the resulting states beyond the idealized picture and their effect on dynamics remains unclear.

In the idealized picture, the coupling of *N* molecules to a single cavity mode leads to the formation of two hybrid light–matter states, the upper and lower polaritons, along with *N* – 1 optically dark states that together form the exciton reservoir.^34,35^ The polaritonic states are delocalized over all molecules coupled to the cavity mode, while each dark state is predominantly localized on an individual molecule.^7–12^ For a long time, the delocalized nature of polaritonic states was believed to be unaffected by inhomogeneous broadening. A pioneering work using a framework similar to the Tavis–Cummings model observed the influence of molecular inhomogeneous broadening on polariton line widths.^36^ This analysis revealed no change in the line width of the polaritonic states but a broadening of the exciton reservoir due to molecular disorder.

Recent studies have challenged the notion that a narrow polariton line width is indicative of a delocalized polaritonic state. It has been shown that the idealized polaritonic picture holds only when the Rabi splitting is significantly larger than the inhomogeneous line width,^37–41^ a condition rarely achieved for organic molecules. When the overlap between the polariton and molecular absorption profiles is significant, states in the exciton reservoir can acquire a partial photonic contribution. These “gray states” bridge the gap between the bright polaritonic and dark exciton reservoir states.^29,38,42,43^ As the number of gray states increases, the photonic content per polaritonic state decreases.^44^ This reduces their delocalized character, extends the polariton lifetime, and opens multiple relaxation pathways.^45^

Despite the significant role that dark and gray states play in polariton photophysics and chemistry, no systematic experimental effort has been made to quantify them. Here, we quantitatively determine the size of the exciton reservoir as a function of detuning using rate equation modeling of emission data. We find that the size of the exciton reservoir and the number of gray states can be controlled by detuning of the cavity mode. Detuning affects the overlap between the molecular and polariton absorption profiles while keeping the collective coupling strength constant. This overlap affects the idealness of the polaritonic system and thus affects the ratio of dark to gray states in the polariton branches. This knowledge can be applied readily to all previous and future studies to interpret how detuning affects the relative number of dark and gray states and, consequently, polaritonic dynamics.

Fabry–Pérot cavities were used to create an optical mode. These cavities consist of two parallel mirrors surrounding an organic molecular layer, and they achieved a quality factor around 60 (Figures 1b, S1, and S2 and supplementary section 1.1). This study presents data from seven such cavities with different energy detuning relative to the excitonic energy. The molecular layer was a neat film of a BODIPY derivative, chosen for its high absorption coefficient and strong fluorescence (QY_em_ = 0.26; Figure 1a and supplementary section 1.2), making it ideal for this study.^46^

**Figure 1:**
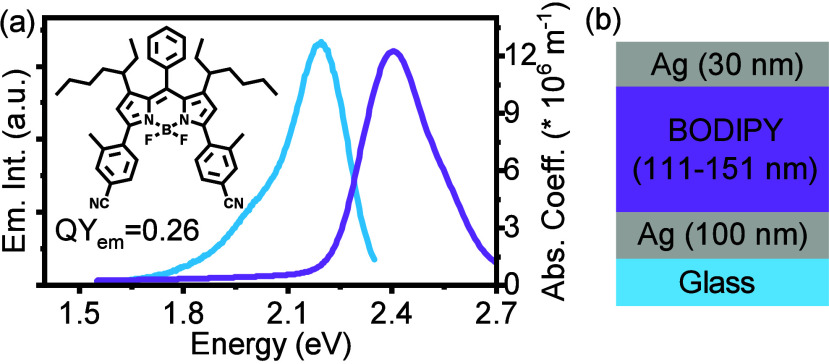
(a) Solid-state absorptivity (purple) and emission (blue) spectra of a pristine film of the BODIPY derivative (processed by spin-coating), along with its molecular structure. (b) Cavity structure from top to bottom: Ag (30 nm), pristine BODIPY film (111–151 nm), Ag (100 nm), glass substrate.

Angle-dependent reflectivity measurements in the transverse electric polarization were performed on all cavities to confirm the establishment of the strong coupling regime. The minima of the angle-dependent reflectivity spectra were fitted using a coupled harmonic oscillator (CHO) model with two excitonic states and one cavity mode (Figure S3, Table S1, and supplementary section 2.1). The total coupling strengths (397–415 meV) were significantly larger than the full-width at half-maximum of both the molecular transitions (222 meV; Figure 1a) and the cavity mode (42 meV; Figure S1), confirming that the cavities were in the strong coupling regime.

The experimental reflectivity data were well reproduced using the transfer matrix method (Figures S4–S12 and supplementary section 2.2), validating that classical electrodynamic simulations capture the main features of the system. Simulations provided the energy dispersion of the cavity modes and the absorption spectrum of the molecular layer within each cavity for later use.

Angle-resolved emission spectra were recorded for both transverse electric (TE) and transverse magnetic (TM) polarizations for each cavity (Figure S13). The lower polariton emission energies coincide with reflectivity minima, confirming their polaritonic origin. To assess the effect of dispersion on relaxation kinetics, the angle-dependent quantum yield of TE-polarized polaritonic emission was determined using polarization-resolved emission spectra and total cavity quantum yields measured with an integrating sphere (Figure S14 and supplementary section 2.3).

Under the assumption that the population transfer from the exciton reservoir into the lower polaritonic states is dominated by radiative pumping,^47^ we constructed a model composed of the lower polariton branch (LP), the exciton reservoir (ER), and the ground state (GS) to simulate the angle-dependent lower polariton emission quantum yield:

$$\frac{d\left[\mathrm{ER}\right]}{dt}=I+\underset{\theta }{\sum }k_{\mathrm{LP}\to \mathrm{ER}}\left(\theta \right)\;\left[\mathrm{LP}\left(\theta \right)\right]-k_{\mathrm{ER}\to \mathrm{GS}}\left[\mathrm{ER}\right]-\underset{\theta }{\sum }k_{\mathrm{ER}\to \mathrm{LP}}\left(\theta \right)\;\left[\mathrm{ER}\right]\mathrm{SA}\left(\theta \;\mathrm{to}\;\theta +5^{{}^{\circ}}\right)$$ 1

and

$$\frac{d\left[\mathrm{LP}\left(\theta \right)\right]}{dt}=k_{\mathrm{ER}\to \mathrm{LP}}\left(\theta \right)\;\left[\mathrm{ER}\right]\mathrm{SA}\left(\theta \;\mathrm{to}\;\theta +5^{{}^{\circ}}\right)-k_{\mathrm{LP}\to \mathrm{GS}}\left(\theta \right)\;\left[\mathrm{LP}\left(\theta \right)\right]-k_{\mathrm{LP}\to \mathrm{ER}}\left(\theta \right)\;\left[\mathrm{LP}\left(\theta \right)\right]$$ 2

Here, [ER] and [LP] represent the populations, *I* is the excitation intensity, and SA is a scaling factor that captures how the area on a hemisphere correlates with the emission angle (measured at 5° intervals; Figure S14 and supplementary section 2.3). The SA was essential in the rate law equations in order to incorporate the polariton density at each emission angle,^48^ which was thus, simplistically, approximated by the area on the hemisphere surface. All pathways are shown in Figure 2a, with *k*_ER→GS_, *k*_ER→LP_, *k*_LP→ER_, and *k*_LP→GS_ the transition rates, as detailed below.

**Figure 2:**
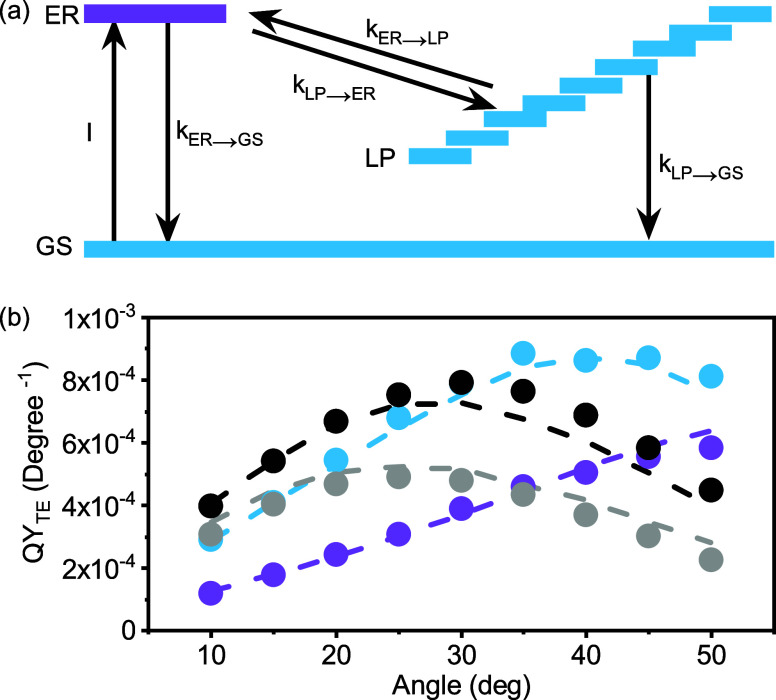
Modeling lower polariton emission quantum yield. (a) Schematic of kinetic models consisting of the exciton reservoir (ER), the lower polariton branch (LP), and the ground state (GS). (b) Circles represent the measured emission quantum yield, and the dashed lines represent the calculated quantum yield using the kinetic model. The purple, blue, black, and gray colors correspond to cavities with thicknesses of 151, 130, 118, and 111 nm, respectively.

The rate for radiative decay from the lower polariton branch was expressed as

3 $$k_{\mathrm{LP}\to \mathrm{GS}}\left(\theta \right)=\frac{E_{c}\left(\theta \right){\left|C_{\mathrm{ph}}\left(\theta \right)\right|}^{2}}{\hbar \mathrm{QF}\left(\theta \right)}$$

with *E*_c_(θ) the cavity mode energy, |*C*_ph_(θ)|^2^ the photonic fraction (Hopfield coefficient) of the lower polariton at angle θ, obtained from fitting the CHO model to reflectivity spectra, and QF­(θ) the quality factor, obtained from Lorentzian fits to the polaritonic emission.

The nonradiative decay rate from the exciton reservoir into the ground state, *k*_ER→GS_, was assumed to be unaffected by the cavity and thus approximated as the rate of a neat BODIPY film outside of the cavity using its emission lifetime (1.29 ns) and quantum yield (0.26).

The rate of back-transfer from the lower polariton branch into the exciton reservoir was modeled as thermally activated and scaled by the excitonic fraction (|*C*_mol_(θ)|^2^):

$$k_{\mathrm{LP}\to \mathrm{ER}}\left(\theta \right)=C_{3}{\left|C_{\mathrm{mol}}\left(\theta \right)\right|}^{2}\int {\mathrm{Abs}}_{\mathrm{film}}\left(E\right)\;e^{-(E-E_{\mathrm{LP}})/k_{B}T}\;$$ 4

with Abs_film_ the area-normalized absorption of a neat BODIPY film (Figure S15 and supplementary section 2.4), *E*_LP_(θ) the energy of the lower polariton at each angle, and *C*_3_ a global fitting parameter. For exothermic back-transfer, the exponential factor was set to unity (Figures S19 and S20 and supplementary section 2.5).

In radiative pumping, excitations within the exciton reservoir relax to the bottom of the molecular S_1_ potential-energy surface prior to emission into the lower polariton.^47,49,50^ As shown in the Supporting Information, the emission from this state into the lower polariton can be described by Fermi’s golden rule (supplementary section 2.6).

5 $$k_{\mathrm{ER}\to \mathrm{LP}}\left(\theta \right)=\frac{2\pi }{\hbar }{\left|g\right|}^{2}\int {\mathrm{Abs}}_{\mathrm{LP}}\left(\theta \right)\;{\mathrm{Em}}_{\mathrm{film}}\left(E\right)\;dE$$

where Abs_LP_(θ) is the optical visibility of the lower polariton (Figures S16 and S17). The optical visibility was measured by 1 – *R*, and it describes (i) the optical accessibility of the lower polariton, (ii) its visibility, (iii) the photonic density of states, and (iv) its spectral overlap with the area normalized film emission (Em_film_; Figures S18). *g* is the single molecular coupling strength, proportional to the transition dipole moment, that couples to the cavity field. As the transition dipole moment is the same for absorption and emission (supplementary section 2.7 and Figure S21), *g* is the same for both processes. Because for Fabry–Pérot cavities |*g*|^2^ is inversely proportional to the cavity mode volume, *g* scales with the cavity length, *L*, as *g* = *g*_0_/√*L*. Therefore, *g*_0_ was used as the second global fitting parameter. Finally, the quantum yield of emission from the lower polariton was calculated as

6 $$\mathrm{QY}\left(\theta \right)=\frac{k_{\mathrm{LP}\to \mathrm{GS}}\left(\theta \right)\;\left[\mathrm{LP}\left(\theta \right)\right]}{I}$$

where *I* is the excitation intensity. The measured lower polariton emission quantum yield was fitted using this kinetic model, with *C*_3_ and *g*_0_ as the global fitting parameters. The experimental and calculated angle-dependent quantum yields for four cavities are shown in Figure 2b (others in Figure S22). The fitting parameters are given in Table S2. The fitted values follow the experimental ones very well, despite only two global fitting parameters. In all cases, the quantum yield increases with the angle until a point where it starts to level off and then decreases. Furthermore, the maximum shows a clear correlation with the cavity thickness, with the most blue-detuned one having the maximum at the lowest angle.

The single-molecule coupling strengths *g* obtained from the *g*_0_ fitting parameter, are plotted in Figure 3a as a function of the cavity length. Because the collective coupling strength *g*_*N*_, which gives rise to the Rabi splitting, scales as *g*_*N*_ = *g*√*N*, the total number of coupled molecules can be computed as

7 $$N=\frac{{g_{N}}^{2}}{g^{2}}$$

Here, *g*_*N*_ was obtained by fitting the reflectivity spectra to the CHO model (Table S1), while *g* was determined from fits to the quantum yield (eq 6 and Table S2).

**Figure 3:**
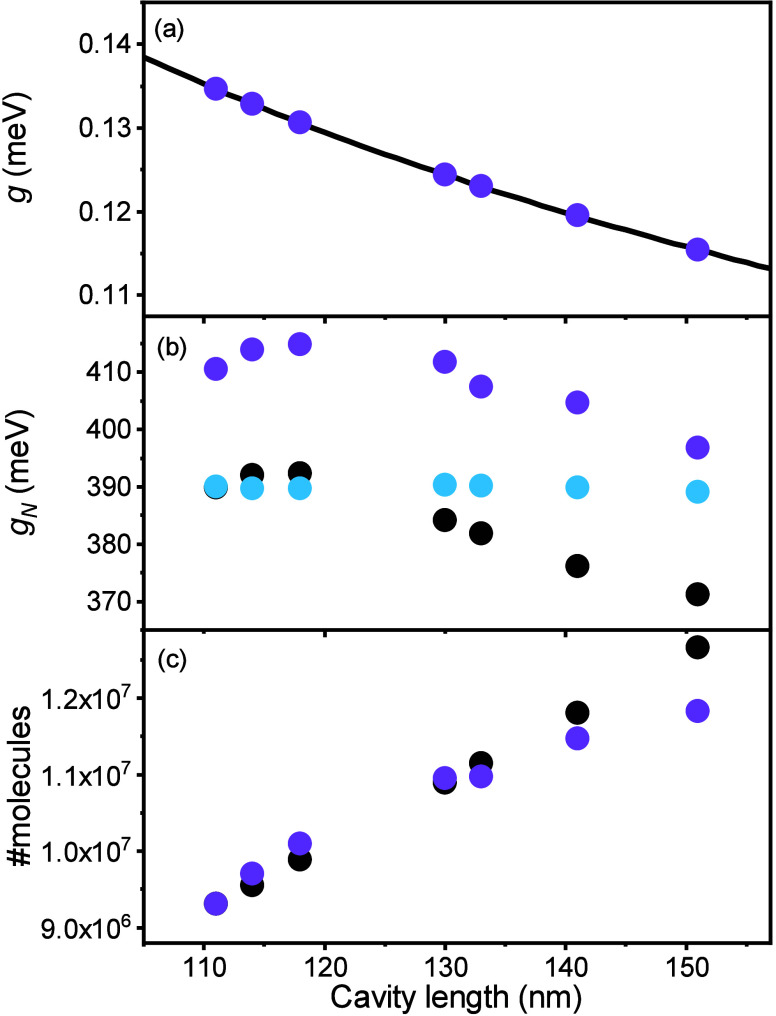
(a) Single-molecule coupling strengths as a function of the cavity length, obtained by fitting the kinetic model to the experimental photoluminescence quantum yield. (b) Collective coupling strengths obtained by fitting the CHO model to experimental (purple) and simulation data with (black) and without (blue) disorder. (c) Number of coupled molecules as a function of the cavity length, using *g*_*N*_ when disorder and disorder-free simulations give the same *g*_*N*_ (black) or *g*_*N*_ obtained from a CHO fit to the reflectivity data (purple).

In principle, the collective coupling strength should be independent of cavity length; however, we observed a small but clear variation (Figure 3, purple circles). To rationalize this behavior, we constructed a microscopic model of the BODIPY-cavity system (supplementary section 2.9) and computed the upper and lower polariton branches both with and without molecular disorder, using the single-molecule coupling strength, *g*, obtained from the rate equation modeling. These branches were subsequently fitted to the CHO model to extract the effective coupling strengths, *g*_*N*_, with and without disorder, which can be directly compared to the expected scaling *g*√*N*. Consistent with the experiments, the fitted *g*_*N*_ values obtained from our disordered cavity models (Figure 3b) vary with the cavity length and follow the same trend. In contrast, in the absence of disorder, the fitted collective coupling strength remains constant and agrees with the input single-molecule coupling strength (Figure 3b).

Following Musser and co-workers,^43^ we attribute this variation to small shifts in the absorption maxima arising from spectral overlap between polaritonic and molecular states. Because the fitted collective coupling strengths are consistent with the microscopic input value for the smallest cavity length, we use this cavity to determine *g*_*N*_ and consequently, the number of coupled molecules in the experiments (Figure 3c). Independently estimating the effective mode volume from the fitted single-molecule coupling strength and the inferred number of coupled molecules, yields consistent values on the order of 10^7^ nm^3^, supporting the internal consistency of the analysis (supplementary section 2.10). Furthermore, taking the cavity specific experimental *g*_*N*_ results in ∼7% deviation from the disorder corrected value.

To summarize, the size of the exciton reservoir can be determined by extracting the microscopic coupling strength from rate equation modeling of emission yields and the collective coupling strength from CHO modeling. Furthermore, care must be taken when determining the collective coupling strength as disorder is affecting values extracted from reflectivity spectra.

In addition to inducing spectral shifts, the energetic overlap between the lower polariton and exciton reservoir reduces the delocalization of the lower polariton states. This effect is quantified by the inverse participation ratio (IPR) of the molecular excitations,^51,52^

8 $$\mathrm{IPR}=\overset{N}{\underset{j=1}{\sum }}{\left|c_{\mathrm{mol},j}\right|}^{4}/{\left(\overset{N}{\underset{j=1}{\sum }}{\left|c_{\mathrm{mol},j}\right|}^{2}\right)}^{2}$$

where |*c*_mol,*j*_|^2^ denotes the contribution of molecule *j* to the polariton state. A fully delocalized polariton, with equal contributions from all molecules, yields IPR = 1/*N*, whereas a fully localized state gives IPR = 1. The microscopic model was used to calculate the IPR. As shown in Figure 4a–c (Figure S23), decreasing the cavity length or increasing the angle enhances the overlap between the lower polariton and molecular states, while simultaneously reducing delocalization.

**Figure 4:**
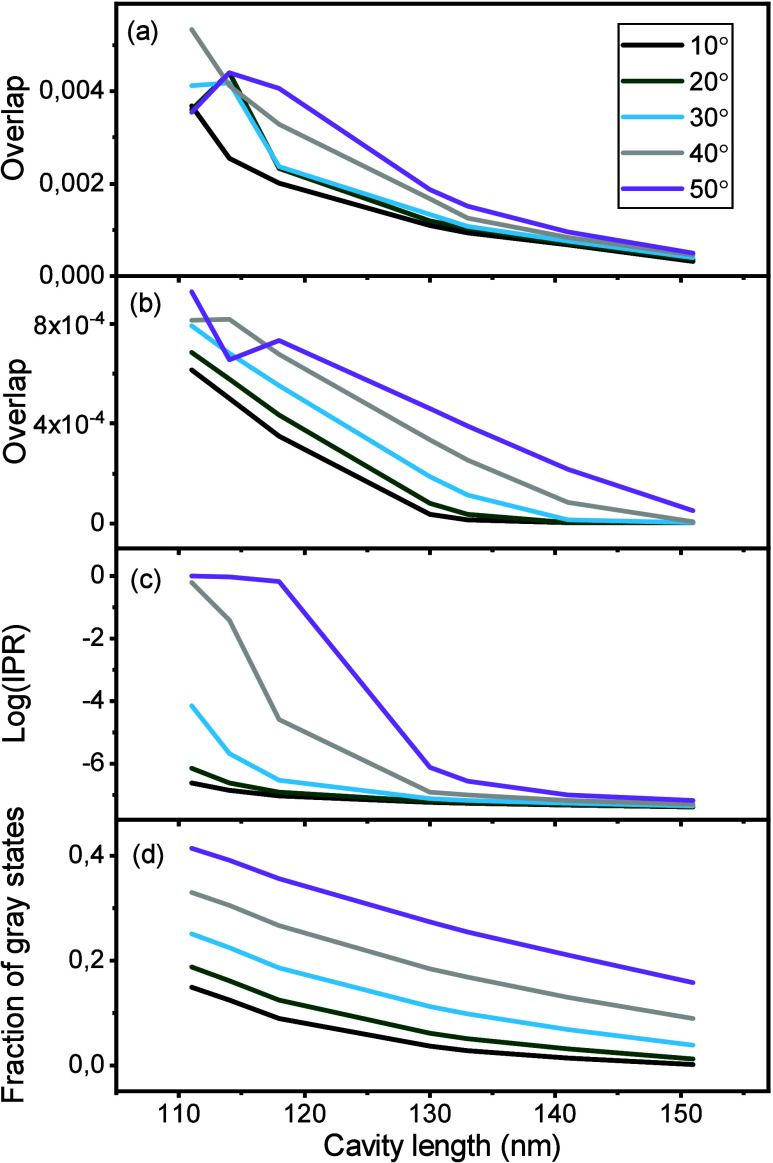
Overlap between the lower polariton and BODIPY film absorption from (a) experiments and (b) microscopic model as a function of the cavity length. (c) Molecular IPR and (d) fraction of gray states as calculated by the microscopic model.

The overlap not only decreases delocalization but also distributes the cavity photon over a larger number of states, leading to so-called gray states.^29,42,44^ Defining the dark state manifold as the set of states that collectively contain less than 10% of the cavity photon, we define gray states as the complement within the (*N* – 1)-state manifold (i.e., the difference between *N* – 1 and the number of dark states). As shown in Figure 4d, the fraction of gray states in the microscopic model follows the same trend as the overlap.

In summary, the microscopic model reproduces the experimentally observed variations in Rabi splitting with the cavity length. These variations can be quantitatively attributed to an increased fraction of gray states, which rises with the spectral overlap between polaritonic and molecular states.

Here, we measured the absolute value of the exciton reservoir (equal to the number of dark states in the idealized picture) in optical cavities of different detuning. To do this, angular resolved emission was coupled with rate equation modeling, with the molecular coupling strength as a global fitting parameter. The size of the exciton reservoir was then correlated through the Rabi splitting. It was found that the value of the exciton reservoir deviates (∼7%) from expectations. This is because the collective coupling strength, as extracted from reflectivity data, becomes slightly off when there is a spectral overlap between the optical visibility of the lower polariton and the absorption of the molecular film. Microscopic modeling indicates that an increased spectral overlap leads to states having a small photonic component; they become gray. Thus, detuning affects the idealness of the polaritons.

These results demonstrate that cavity detuning does more than tune the photonic and excitonic fractions of polaritons. It also modifies the degree of ideality of the polaritonic system, thereby affecting fundamental properties such as transition pathways and polariton lifetimes. Moreover, the experimental access to the size of the exciton reservoir established here provides a new route to quantitatively characterize polaritonic systems. In particular, it enables the back-extraction of effective mode volumes and offers a benchmark for developing and validating simulation approaches capable of describing the collective coupling of large molecular ensembles.^53^

## Supplementary Material



## Data Availability

Raw data are deposited at the Swedish National Data Service at https://doi.org/10.5878/fjnv-d614.

## Notes

Additional experimental details, analyses, and materials and methods, along with additional spectroscopic and simulated data (PDF)
