# Supplementary material for: Quantifying the Number of Dark, Gray, and Bright States as a Function of the Spectral Overlap in Polaritonic Systems
Source: Nano Lett. 2026 May 27;26(22):7257–62. doi: 10.1021/acs.nanolett.5c06424 (PMC13267169; doi:10.1021/acs.nanolett.5c06424)
Supplement: Supplementary file 1 [file nl5c06424_si_001.pdf]

Supplementary Information for

Quantifying the Number of Dark, Gray, and Bright States as a Function of Spectral Overlap in Polaritonic Systems

Rahul Bhuyan[a], Ilia Sokolovskii[b], Clara Schäfer[a], Gerrit Groenhof[b,*], Karl Börjesson[a,*]

[a]Department of Chemistry and Molecular Biology, University of Gothenburg, Gothenburg, 41390, Sweden.
[b]Nanoscience Center and Department of Chemistry, University of Jyväskylä, 40014 Jyväskylä, Finland

*Corresponding Author: karl.borjesson@gu.se

Contents

1. Experimental Methods 2

1.1. Cavity Preparation 2

1.2. Steady State Absorption, Reflection, and Emission Spectroscopy 2

2. Supplementary Figures and Analysis 3

2.1. Coupled harmonic oscillator model (including Figure S3) 4

2.2. TMM calculation (including Figures S4-12) 7

2.3. Calculation of the QY at each angle (including Figures S13-14) 11

2.4. The absorption of the lower polariton (Abs_{LP}) and the neat film (Abs_{film}) (including Figures S15-17) 15

2.5. Calculation of the integrated part of $k_{ER\rightarrow LP}$, and $k_{LP\rightarrow ER}$ (including Figures S18-20) 17

2.6. Motivation why the transition into the lower polariton follow the Fermi's golden rule 19

2.7. Electronic structure calculations of BODIPY (including Figure S21) 21

2.8. Figure S22 22

2.9. Microscopic modeling 23

2.10. An approximation of the number of molecules in the mode volume 25

2.11. Calculation of the overlap between the absorption of the LP and the film (including Figure S23) 26

2.12. Figure S24 27

3. Supplementary Tables 28

4. References 29

1. Experimental Methods

1.1. Cavity Preparation

2.5 cm×2.5 cm glass substrates (microscope slide purchased from J. Melvin Freed) underwent a 15-minute sonication in a 1% alkaline solution (Hellmanex in Milli-Q water). Subsequently, glass slides were sonicated for 1 hour in Milli-Q water and then ethanol. After the cleaning process, the glass substrates were dried in an oven overnight before the fabrication of films or cavities. The Ag mirrors were produced through vacuum sputtering deposition (HEX from Korvus Technologies). Initially, a 100 nm Ag mirror was sputtered onto the glass substrate. Subsequently, 100 µl of a solution of the BODIPY derivative (36 mg/ml in toluene) was spin-coated at 1400 to 2800 rpm for 1 minute on the surface of the Ag mirror. The synthesis of the BODIPY derivative was done in accordance to the literature.[1] The films were then incubated for four days to reach a stable emission quantum yield with time. To complete the optical cavity, a semi-transparent 30 nm Ag mirror was sputtered onto the molecular film. The reflectivity and transmittance spectra of 30 nm Ag mirror shown in Figure S2.

1.2. Steady State Absorption, Reflection, and Emission Spectroscopy

Steady-state absorption of films and reflectivity of cavities were recorded using a Perkin Elmer LAMBDA 950 spectrometer. Angle-dependent reflectivity of cavities was measured by a Universal Reflectance Accessory (URA, Perkin Elmer). Spectra were referenced to a standard reflectance mirror inside the URA, employing a Glan-Taylor polarizer. Reflectivity spectra were collected in transverse electric (TE) polarization. The solid-state absorption spectrum of the BODIPY derivative has one prominent transition at 2.379 eV and a vibronic shoulder at 2.543 eV.

Steady-state emission spectra of the films and cavities were measured on an Edinburgh Instruments FLS 1000 spectrofluorometer, with a Xenon lamp as the excitation source and a double monochromator for wavelength selection (3 nm slit sizes for both excitation and emission). The emission spectrum is a mirror image of the absorption spectrum, with the primary transition at 2.196 eV and the vibronic shoulder at 2.02 eV. The solid-state emission quantum yield is 0.26 four days after spin-coating, which is notably high for pristine films of organic dyes (Figure 1a). Angle-resolved emission spectra of the cavities were measured for TE, and TM polarization on the same instrument. This was achieved through a liquid lightguide connected to an angle-resolved platform and using a polarizer in the emission pathway. To measure the emission spectra, the exciton reservoir was excited at 2.398 eV and at a 15-degree angle normal to the surface. Emission from the cavities was recorded at angles ranging from 10 to 50 degrees in 5-degree intervals. Excitation and emission angles were kept orthogonal to each other to prevent specular reflection into the detector. The lower polaritonic emission quantum yield of all the cavities was measured using integrating sphere.

During the conversion of the emission spectra from a wavelength (nm) scale to an energy (eV) scale, the intensity was scaled with the square of the wavelength as described in the following reference.[2]

2. Supplementary Figures and Analysis

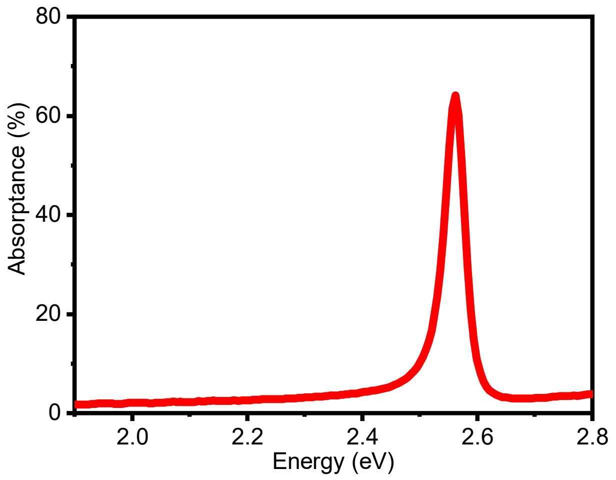

Figure S1. Absorptance spectrum (measured as 1-reflectivity) of an empty cavity (filled with PVA) with the same structure as cavities containing the BODIPY derivative. The full width at half maximum (FWHM) of the cavity mode is 42 meV, corresponding to a quality factor of 60.

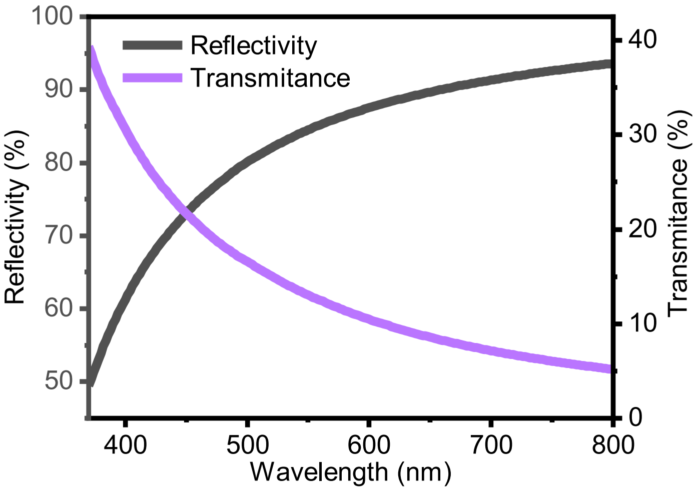

Figure S2. Reflectivity (black) and transmittance (purple) spectra of a 30 nm Ag mirror measured at 10 degrees.

2.1. Coupled harmonic oscillator model (including Figure S3)

The cavity mode energies extracted from the TMM fitting (see next section) were applied in the coupled harmonic oscillator (CHO) modeling.[3] This approach has the advantage of not requiring parameters like the refractive index, and cavity thickness, as these are accounted for in the TMM calculations. The CHO model was performed by considering two excitonic states along with one cavity mode, assuming no interaction between the excitonic states:

$$\begin{bmatrix} E_{X_1} & 0 & g_1 \\ 0 & E_{X_2} & g_2 \\ g_1 & g_2 & E_C(K_{\parallel}) \end{bmatrix} * \begin{bmatrix} \propto \\ \beta \\ \gamma \end{bmatrix} = E(K_{\parallel}) * \begin{bmatrix} \propto \\ \beta \\ \gamma \end{bmatrix} \tag{S1}$$

Here, E represents the energies of hybrid polaritonic states. E_{X1} (2.379 eV) and E_{X2} (2.543 eV) are the energies of the two vibronic transitions of the molecule. E_C denotes the cavity mode energy, extracted from the TMM calculations. g_1 is the coupling strength between the E_{X1} and E_C, and g_2 is the coupling strength between E_{X2} and E_C. The $|\propto|^2$, $|\beta|^2$, and $|\gamma|^2$ represent the fractional contribution of E_{X1}, E_{X2}, and E_C to the corresponding polaritonic states. For these cavities, g_1 ranged from 171 to 226 meV, and g_2 ranged from 184 to 227 meV (Table S1). The total coupling strengths (g_N) were calculated as $g_1 + g_2$.

The CHO model of polariton dispersion and corresponding Hopfield coefficient of the lower polariton of seven cavities are shown in Figure S3. The Hopfield coefficients corresponding to the cavity mode and excitonic states for the lower polariton were obtained from the CHO model and later used for kinetic modeling of the lower polariton emission quantum yield.

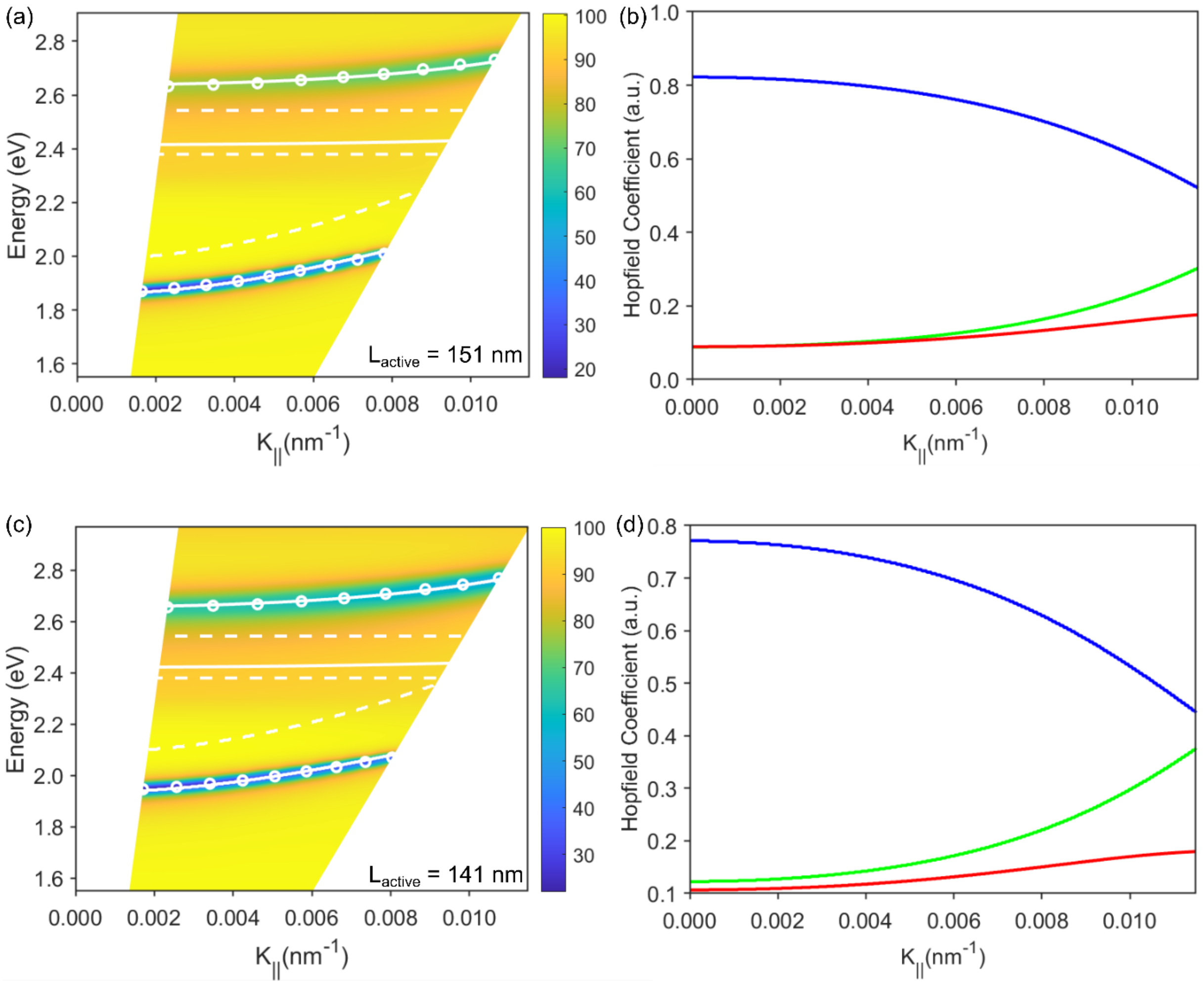

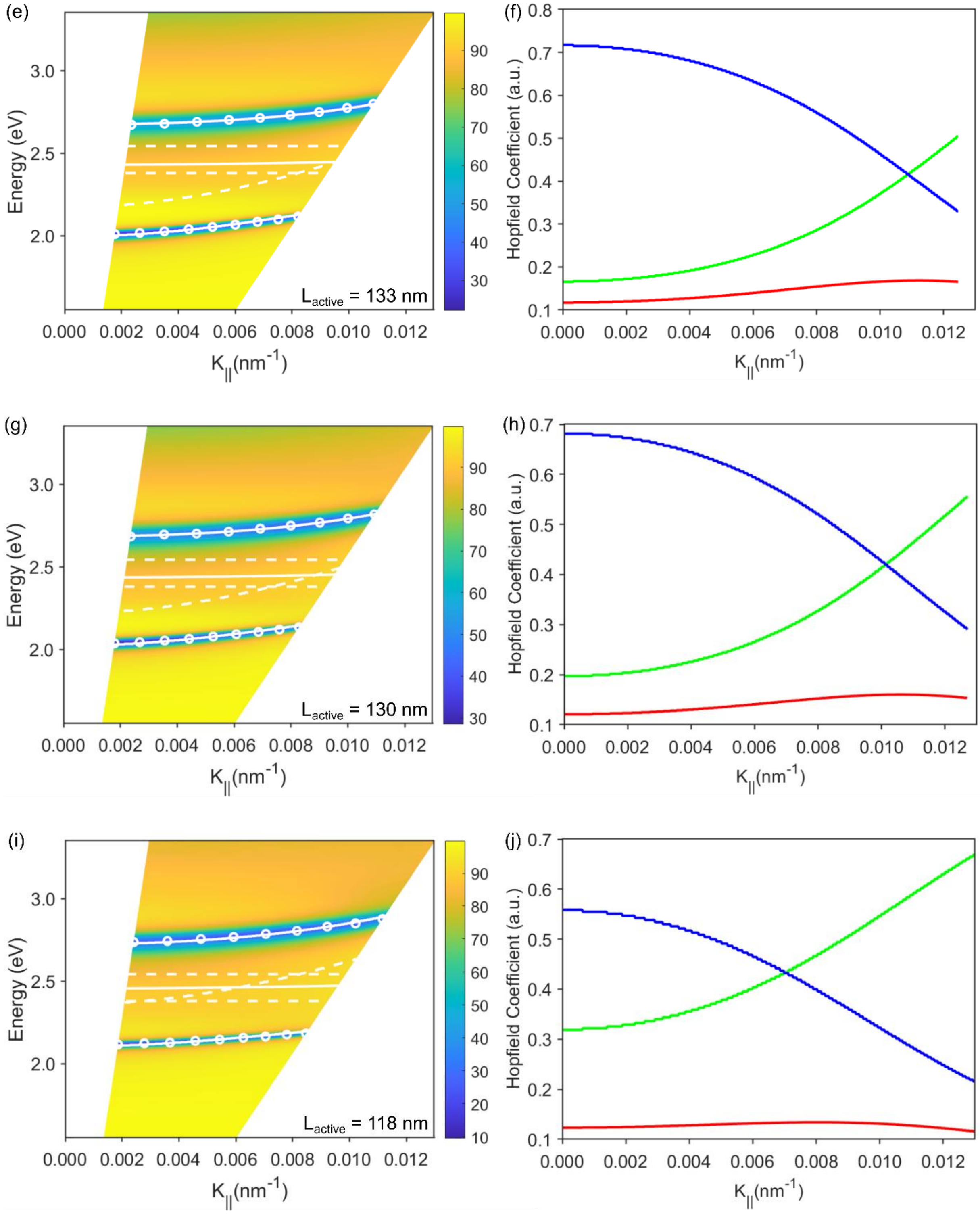
(e)
(f)
(g)
(h)
(i)
(j)
Energy (eV)
Hopfield Coefficient (a.u.)
$K_{||}(nm^{-1})$
L_{active} = 133 nm
L_{active} = 130 nm
L_{active} = 118 nm

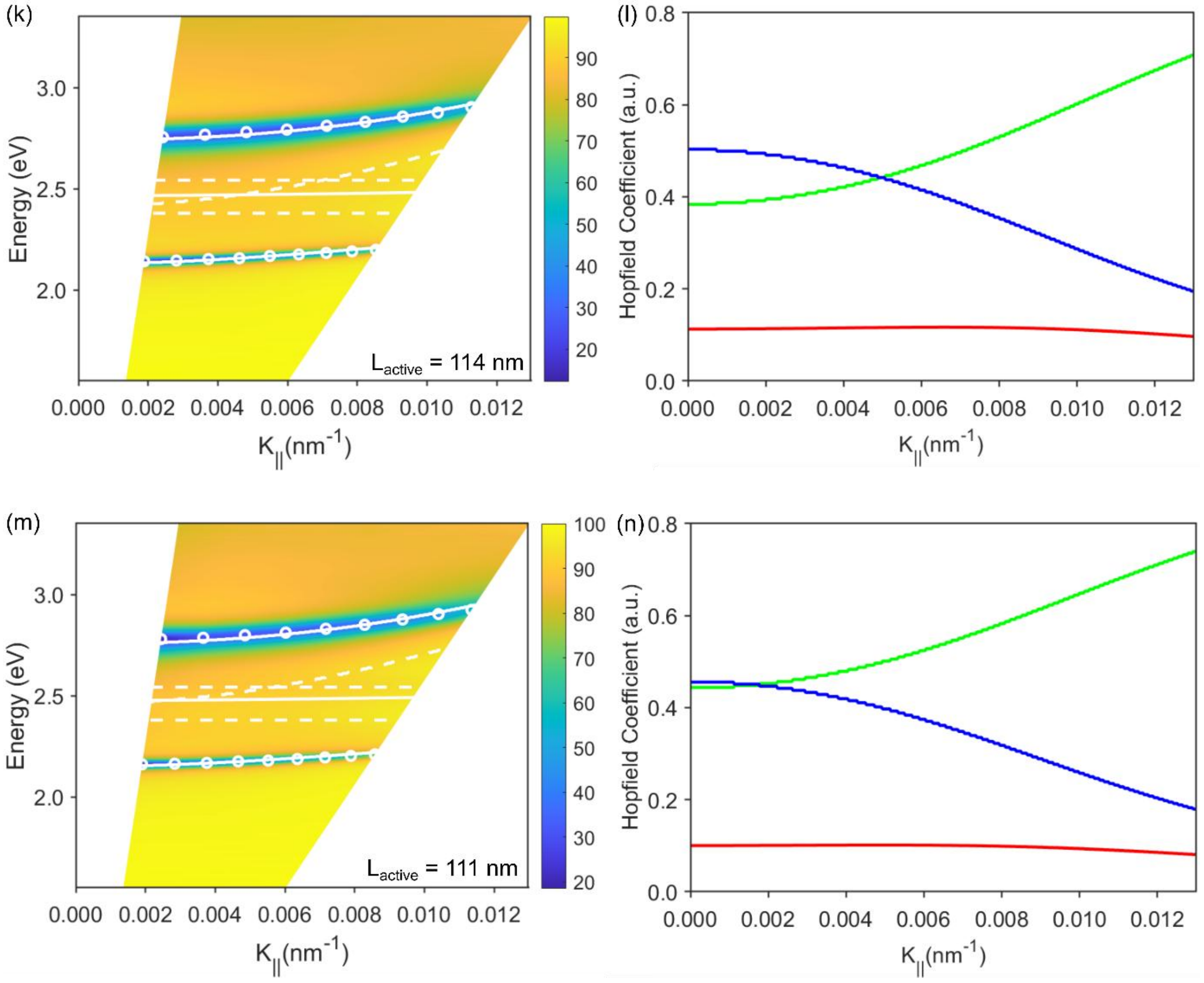

Figure S3. Angle-resolved reflectivity of cavities is shown alongside the lower and upper polariton dispersion, fitted using the coupled harmonic oscillator model. The cavity thicknesses are (a) 151 nm, (c) 141 nm, (e) 133 nm, (g) 130 nm, (i) 118 nm, (k) 114 nm, and (m) 111 nm. The solid white lines represent the fit of the polaritonic dispersion obtained from the coupled harmonic oscillator model, while the dotted lines indicate the energies of the molecular transitions and the cavity mode. The Hopfield coefficients for the lower polariton in cavities with thicknesses of (b) 151 nm, (d) 141 nm, (f) 133 nm, (h) 130 nm, (j) 118 nm, (l) 114 nm, and (n) 111 nm are also displayed. Blue indicates the cavity mode contribution, green shows the contribution from exciton 1 at 2.379 eV, and red corresponds to the contribution from exciton 2 at 2.543 eV.

2.2. TMM calculation (including Figures S4-12)

The experimental reflectivity of all seven cavities was analyzed using a transfer matrix method (TMM) fitting procedure.[4] This process involved adjusting 20 unknown parameters, with six of these corresponding to the dielectric permittivity of Ag, modeled by the Drude-Lorentz equation:

$$\epsilon(\omega) = \epsilon_{\infty} - \frac{f_0^2}{\omega(\omega+i\gamma)} + \frac{f_1^2}{\omega_1^2-\omega^2-i\omega\Gamma} \quad \text{(S2)}$$

where ϵ_{∞}, f_0, γ, f_1, ω_1 and, Γ are fitting parameters. Additional free parameters included the thicknesses of the front mirror and the molecular layer, both specific to each cavity, bringing the total number of parameters to 14. The back mirror thickness was fixed at 100 nm. The dielectric permittivity of the active layer, obtained from ellipsometry measurements, is shown in Figure S4. The refractive index was obtained by fitting 4-voight functions to the n,k data from the ellipsometry measurements. Figure S5 compares the dielectric permittivity of Ag derived from fitting the experimental cavity reflectivity to the standard Drude model for Ag.[5] Figures S6–S12 display the reflectivity profiles generated by the TMM fitting.

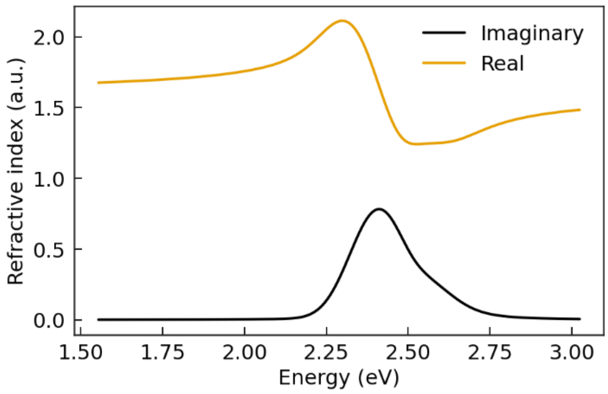

Figure S4. Real (yellow) and imaginary (black) parts of the refractive index of a neat BODIPY film measured using ellipsometry.

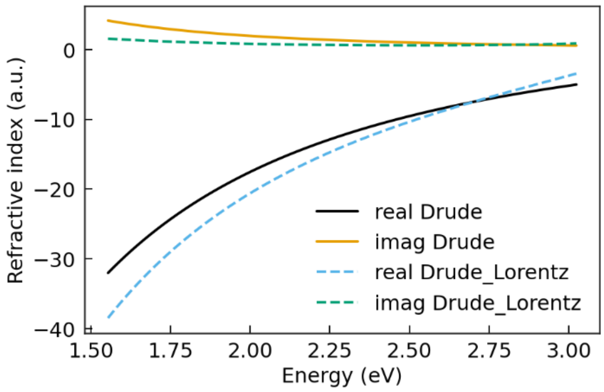

Figure S5. Refractive index of Ag (dotted line) extracted from a TMM calculation using the Drude–Lorentz model.

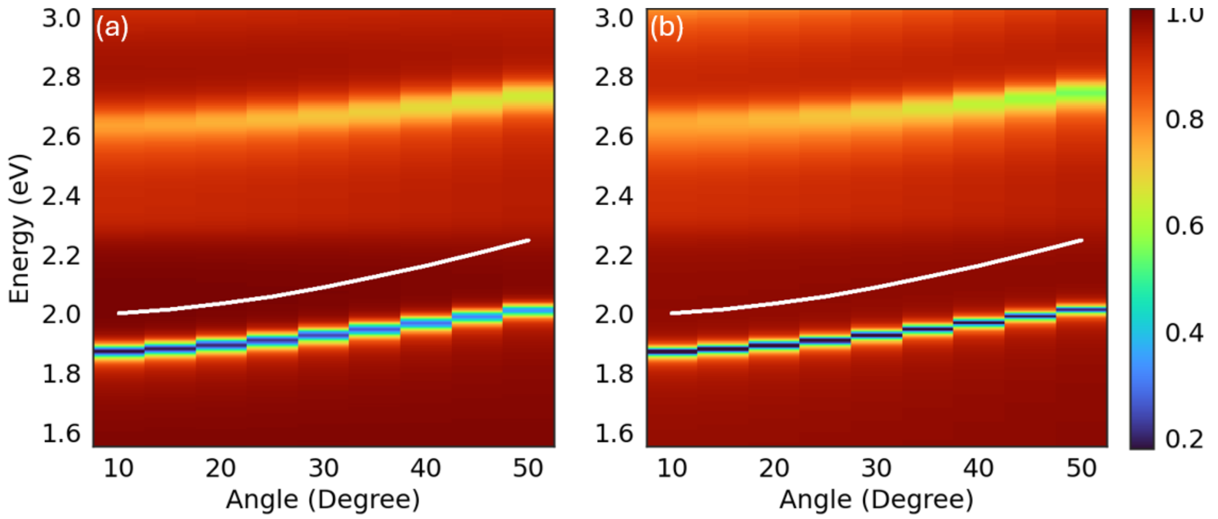

Figure S6. Reflectivity of the cavity with a thickness of 151 nm. (a) Experimental reflectivity of the cavity, and (b) the corresponding TMM-calculated reflectivity. The white solid curve corresponds to the cavity mode dispersion.

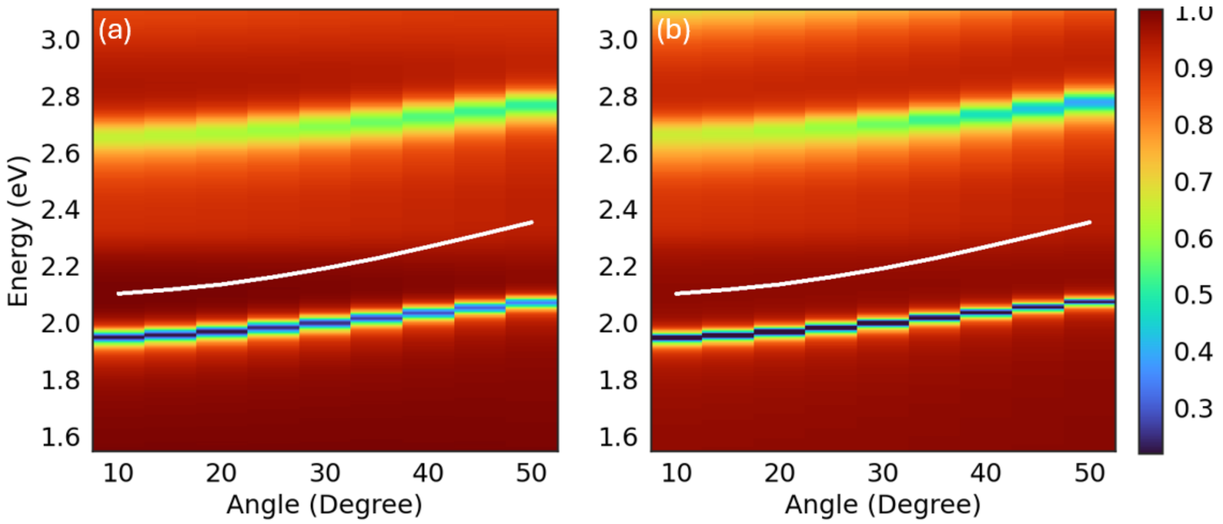

Figure S7. Reflectivity of the cavity with a thickness of 141 nm. (a) Experimental reflectivity of the cavity, and (b) the corresponding TMM-calculated reflectivity. The white solid curve corresponds to the cavity mode dispersion.

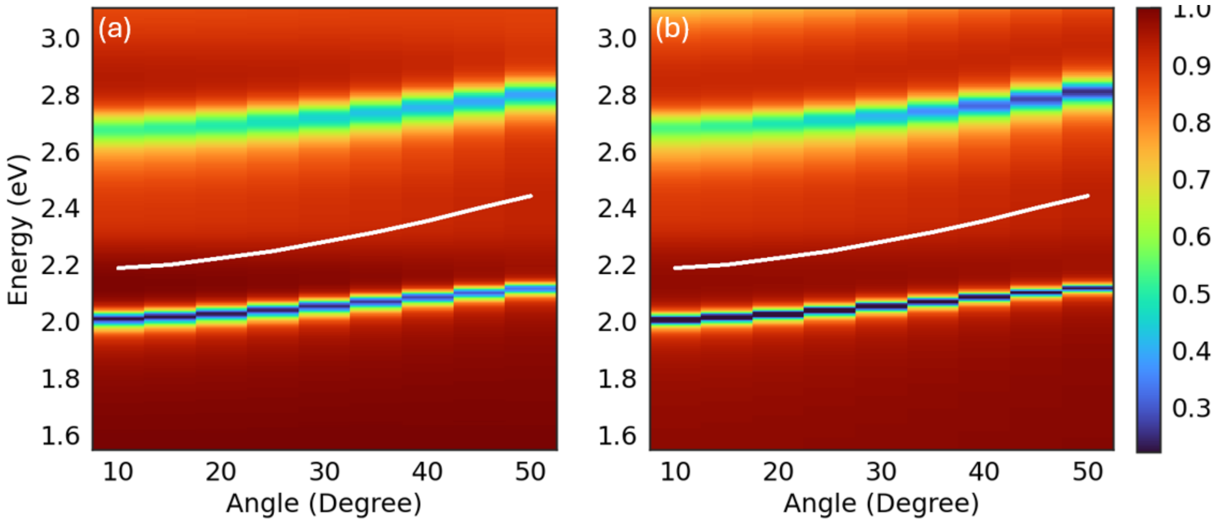

Figure S8. Reflectivity of the cavity with a thickness of 133 nm. (a) Experimental reflectivity of the cavity, and (b) the corresponding TMM-calculated reflectivity. The white solid curve corresponds to the cavity mode dispersion.

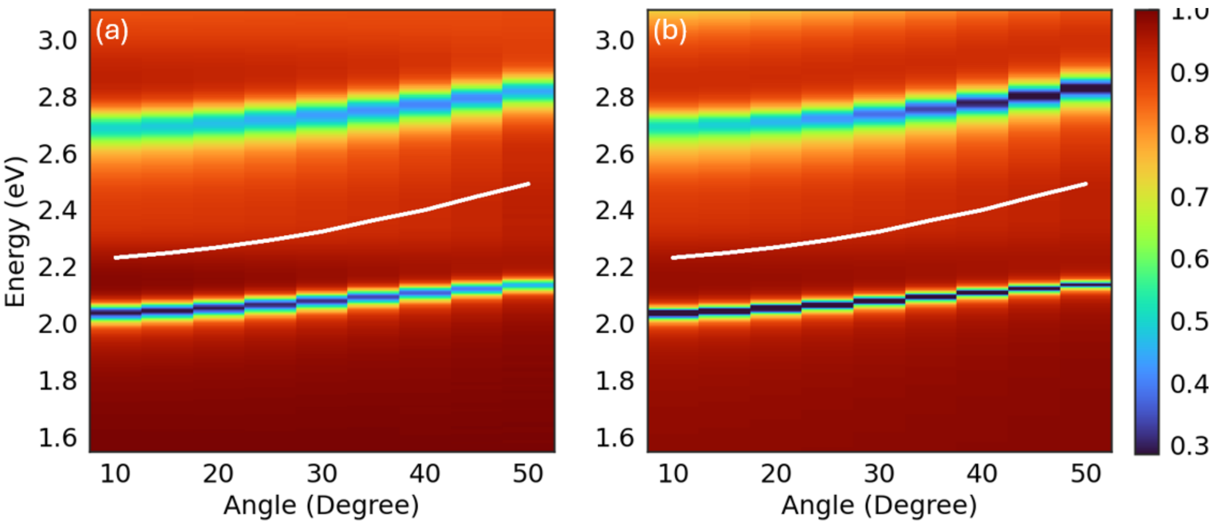

Figure S9. Reflectivity of the cavity with a thickness of 130 nm. (a) Experimental reflectivity of the cavity, and (b) the corresponding TMM-calculated reflectivity. The white solid curve corresponds to the cavity mode dispersion.

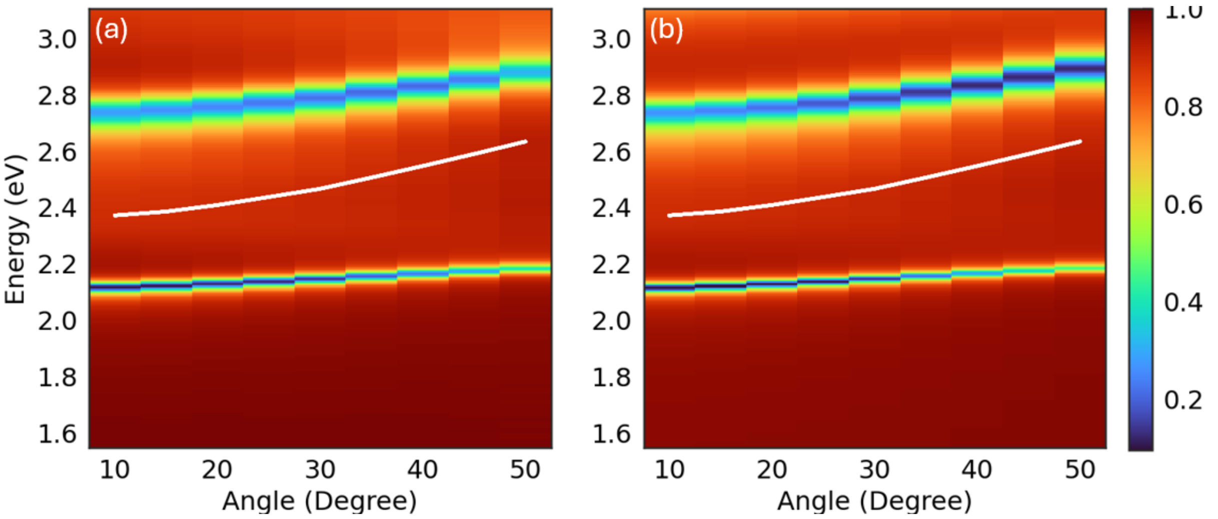

Figure S10. Reflectivity of the cavity with a thickness of 118 nm. (a) Experimental reflectivity of the cavity, and (b) the corresponding TMM-calculated reflectivity. The white solid curve corresponds to the cavity mode dispersion.

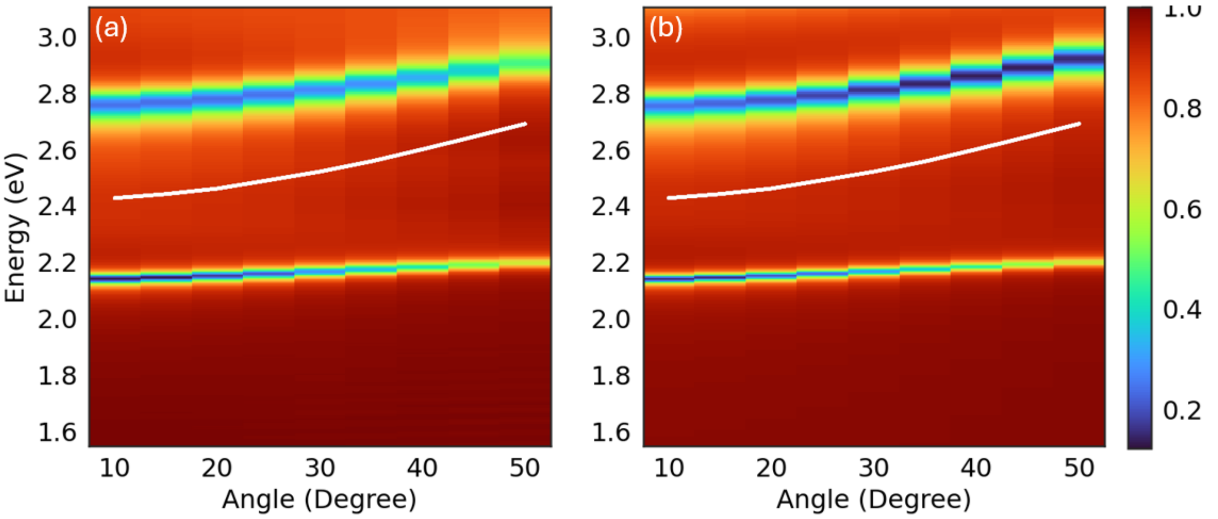

Figure S11. Reflectivity of the cavity with a thickness of 114 nm. (a) Experimental reflectivity of the cavity, and (b) the corresponding TMM-calculated reflectivity. The white solid curve corresponds to the cavity mode dispersion.

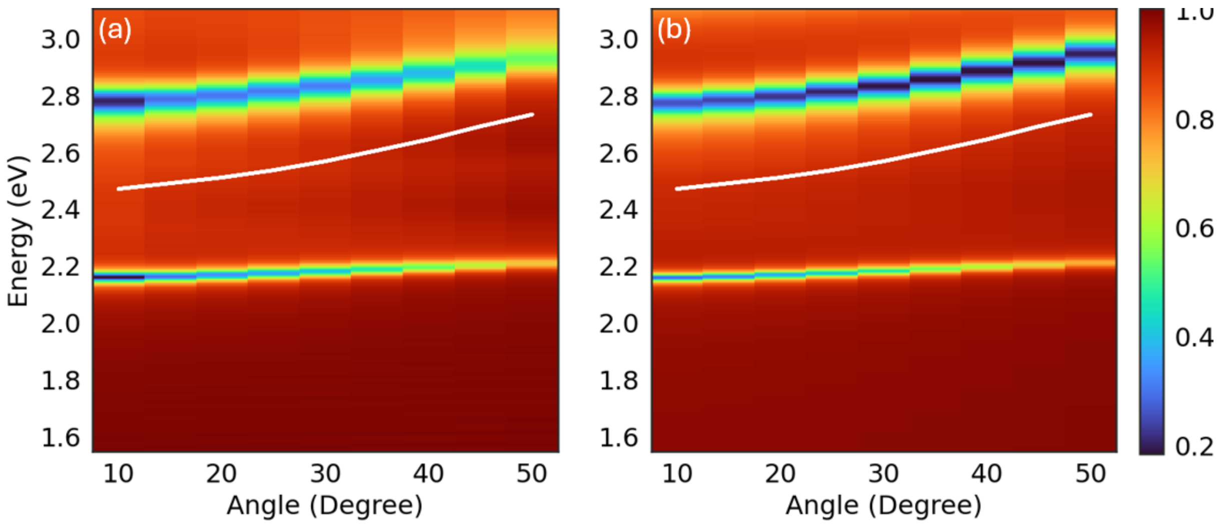

Figure S12. Reflectivity of the cavity with cavity a thickness of 111 nm. (a) Experimental reflectivity of the cavity, and (b) the corresponding TMM-calculated reflectivity. The white solid curve corresponds to the cavity mode dispersion.

2.3. Calculation of the QY at each angle (including Figures S13-14)

In an integrating sphere, the measured quantum yield (QY) corresponds to emission coming out from one side of cavity. Therefore, the total emission was considered to be in the upper hemisphere, assuming a uniform density of polaritonic states across the hemispherical surface (Figure S14). To obtain the QY at each angle, emission intensity for both TE and TM polarizations were measured from 10 to 50 degrees in 5 degree intervals in a quarter circular geometry. To obtain the total emission corresponding to each angle, it needs to be scaled with the surface area of circular ring. The surface area of a ring between angles θ and θ+dθ is given by:

$$\text{Surface area of ring} = 2\pi r * ds = 2\pi R * \cos\theta * R * d\theta \qquad \text{(S3)}$$

For a ring covering an angular range from θ_1 to θ_2, the surface area is:

$$2\pi R^2 \int_{\theta_1}^{\theta_2} \cos\theta * d\theta = 2\pi R^2 \, [\sin\theta_2 - \sin\theta_1] \qquad \text{(S4)}$$

In terms of our emission measurement angle, φ=90-θ, where sin(90-φ) = cosφ, the surface area of a ring spanning φ_1 to φ_2 is:

$$2\pi R^2 \, [\cos\varphi_2 - \cos\varphi_1] \qquad \text{(S5)}$$

To represent this as a fraction of the total hemispherical surface area between φ_1 and φ_2, $SA(\varphi_1 \; to \; \varphi_2)$ is defined as:

$$SA(\varphi_1 \; to \; \varphi_2) = \frac{\text{Surface area of ring between } \varphi 1 \text{ and } \varphi 2}{\text{Total surface area of hemisphere}} = [\cos\varphi_2 - \cos\varphi_1] \qquad \text{(S6)}$$

The total emission intensity between angles φ_1 and φ_2 after scaling with the surface area of the ring, $I(\varphi_1 \; to \; \varphi_2)$, is given by:

$$I(\varphi_1 \; to \; \varphi_2) = (\text{Measured emission intensity at } \varphi_1) * SA(\varphi_1 \; to \; \varphi_2) \qquad \text{(S7)}$$

The overall emission from the cavity was obtained by summing over all angles:

$$\text{Total emission} = \sum_{\varphi_1,\varphi_2} I(\varphi_1 \; to \; \varphi_2) \qquad \text{(S8)}$$

Note: The measured TM emission was corrected for detector sensitivity, calculated as the wavelength-dependent factor obtained by dividing the TE and TM polarized emissions of BODIPY in toluene solution. The BODIPY molecules in the toluene solution rotate faster than the excited state lifetime. So, the transition dipole moment of BODIPY in solution is oriented randomly after excitation. Thus, no anisotropy in the emission from the molecule itself is expected, any seen is due to differences in sensitivity of TE and TM polarized light in the detector channel. Since the integrating sphere measures both TE and TM polarized emissions, the total quantum yield for the TE polarization was calculated as follows:

$$QY_{TE} = \frac{\sum_{\varphi_1,\varphi_2} I_{TE}(\varphi_1 \; to \; \varphi_2) * QY}{\sum_{\varphi_1,\varphi_2} I_{TE}(\varphi_1 \; to \; \varphi_2) + \sum_{\varphi_1,\varphi_2} I_{TM}(\varphi_1 \; to \; \varphi_2)} \qquad \text{(S9)}$$

where $I_{TE}(\varphi_1 \; to \; \varphi_2)$ and $I_{TM}(\varphi_1 \; to \; \varphi_2)$ are the total TE and TM polarized emissions between φ_1 and φ_2, and QY is the measured total quantum yield of the cavity in the integrating sphere. To determine the QY at each circular ring, $QY_{TE}(\varphi_1 \; to \; \varphi_2)$, the following formula was used:

$$QY_{TE}(\varphi_1 \; to \; \varphi_2) = \frac{I_{TE}(\varphi_1 \; to \; \varphi_2) * QY_{TE}}{\sum_{\varphi_1,\varphi_2} I_{TE}(\varphi_1 \; to \; \varphi_2)} \qquad \text{(S10)}$$

The emission intensity that is in the summations of Equations S9 and S10 includes the hemisphere area (according to equation S7). Thus, these summations describe the integrated emission over the entire hemisphere.

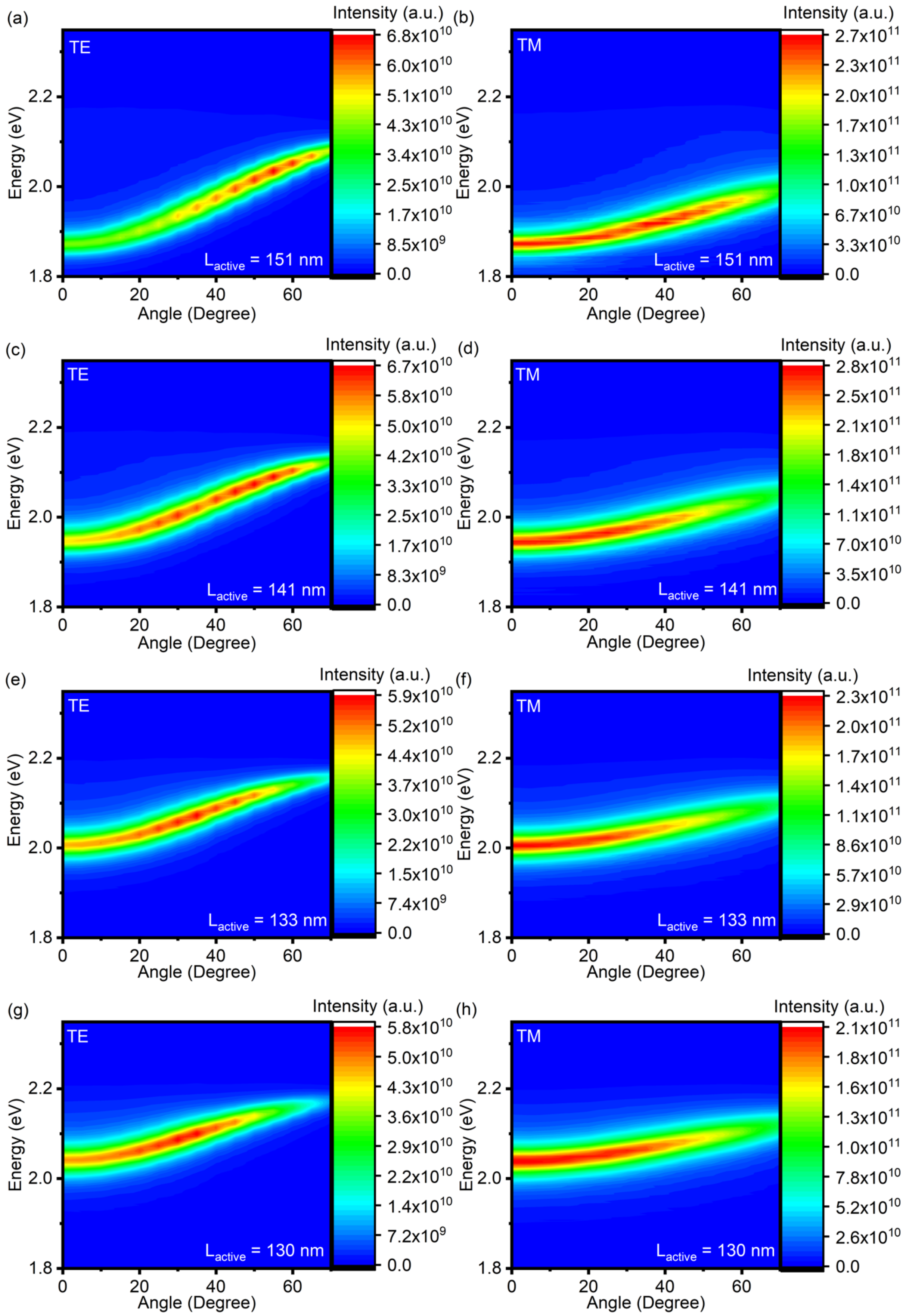
(a)
TE
L_active = 151 nm
Energy (eV)
Angle (Degree)
Intensity (a.u.)
(b)
TM
L_active = 151 nm
(c)
TE
L_active = 141 nm
(d)
TM
L_active = 141 nm
(e)
TE
L_active = 133 nm
(f)
TM
L_active = 133 nm
(g)
TE
L_active = 130 nm
(h)
TM
L_active = 130 nm

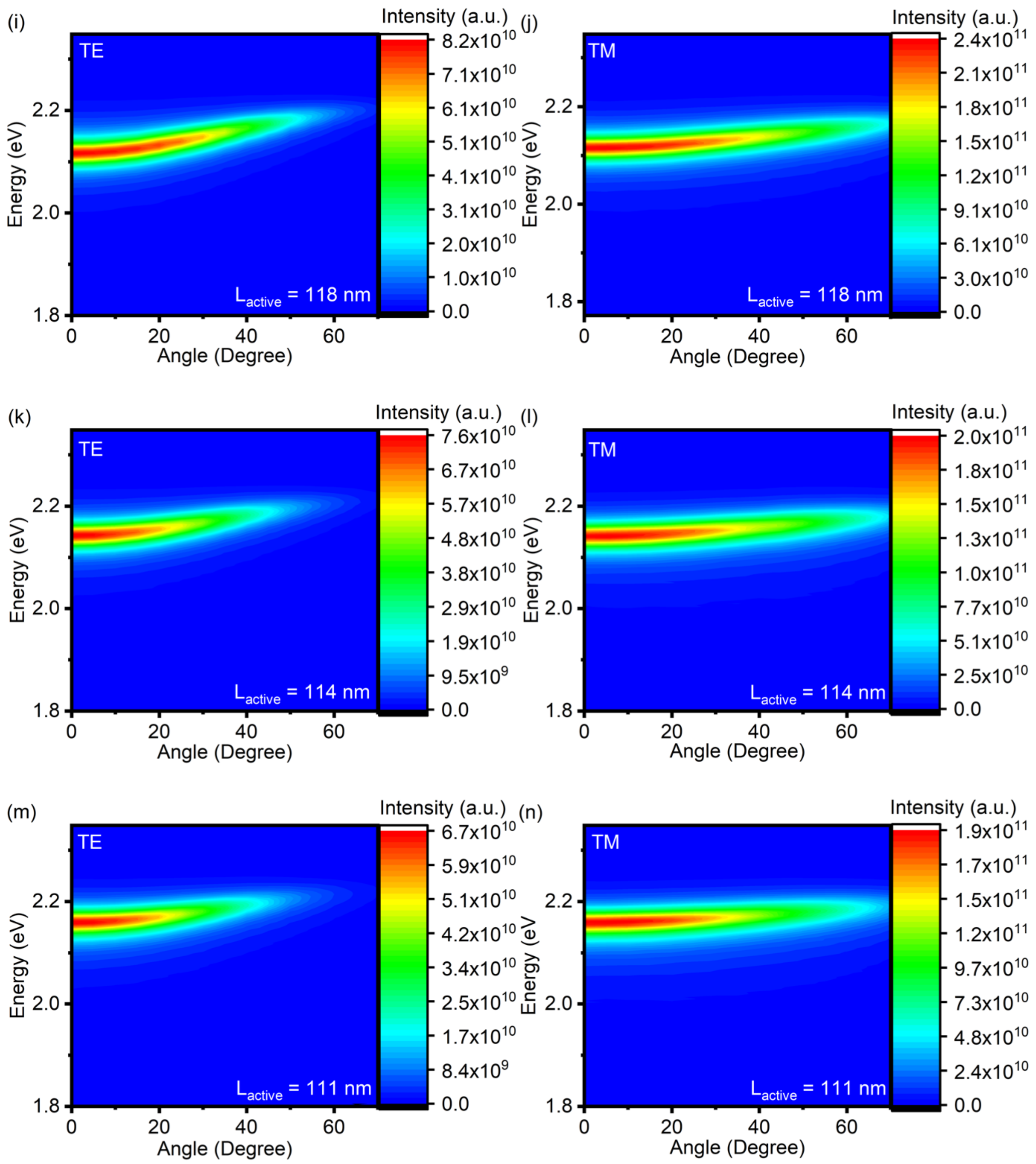

Figure S13. Angle-dependent emission maps of cavities excited at 2.398 eV. Emission recorded with TE polarization corresponds to cavities with thicknesses of (a) 151 nm, (c) 141 nm, (e) 133 nm, (g) 130 nm, (i) 118 nm, (k) 114 nm, and (m) 111 nm. Emission recorded with TM polarization corresponds to cavities with thicknesses of (b) 151 nm, (d) 141 nm, (f) 133 nm, (h) 130 nm, (j) 118 nm, (l) 114 nm, and (n) 111 nm.

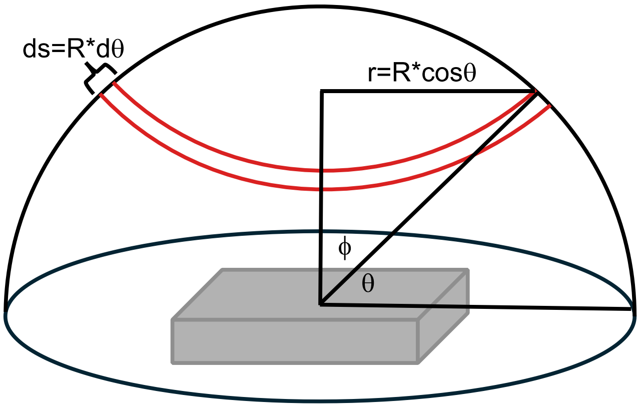

Figure S14. Hemispherical consideration of emission from the cavity. It is assumed that the polariton states have a uniform density over the hemisphere surface. Here, ϕ represents the angle of polariton emission measurements, while ds denote the area of the circular ring corresponding to each angle. R is the radius of the hemisphere.

2.4. The absorption of the lower polariton (Abs_{LP}) and the neat film (Abs_{film}) (including Figures S15-17)

The spectra denoted as Abs_{film} were calculated using TMM for each cavity thickness. This was done to avoid effects from scattering and reflections at interfaces in the data. The Abs_{film} spectra were area normalized and the one representing a 151 nm thick film is shown in Figure S15.

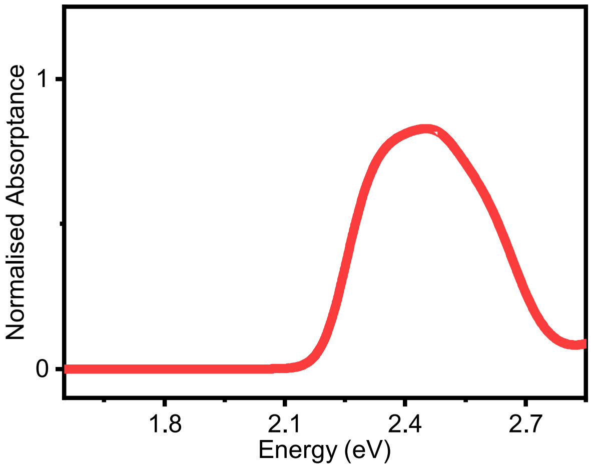

Figure S15. Abs_{film}, which is the area normalized absorptance spectrum, calculated from TMM for in this case a 151 nm thick BODIPY film.

The Abs_{LP} was based on the experimental reflectivity data presented in Figure S3. It was assumed that no light transmits through the thick (100 nm) back mirror, and that the absorption then can be calculated by 1-R. To extract the absorption from the lower polariton, a triple Lorentzian function was fitted to the low energy peak of the 1-R spectra. An example of this fit is shown in Figure S16, and a set of full data over all angles for one cavity is shown in Figure S17.

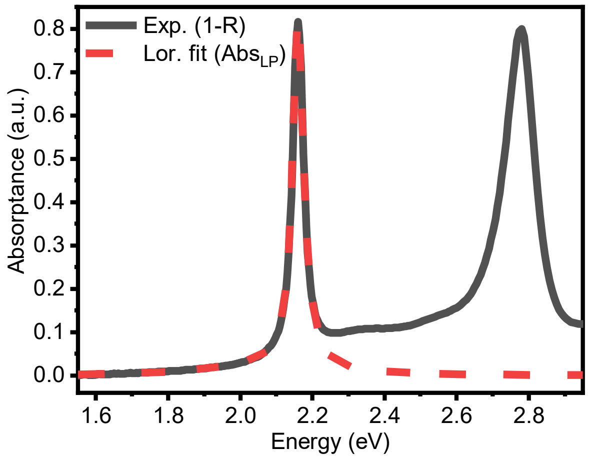

Figure S16. Extraction of the absorptance spectrum of the lower polariton (the optical visibility of the lower polariton, Abs_{LP}) by fitting a triple Lorentzian function to the experimental 1-R spectra. Displayed here for the cavity with a thickness of 111 nm at 10 degrees, which is the most blue-detuned cavity having the largest overlap between the absorption of the lower polariton and other states.

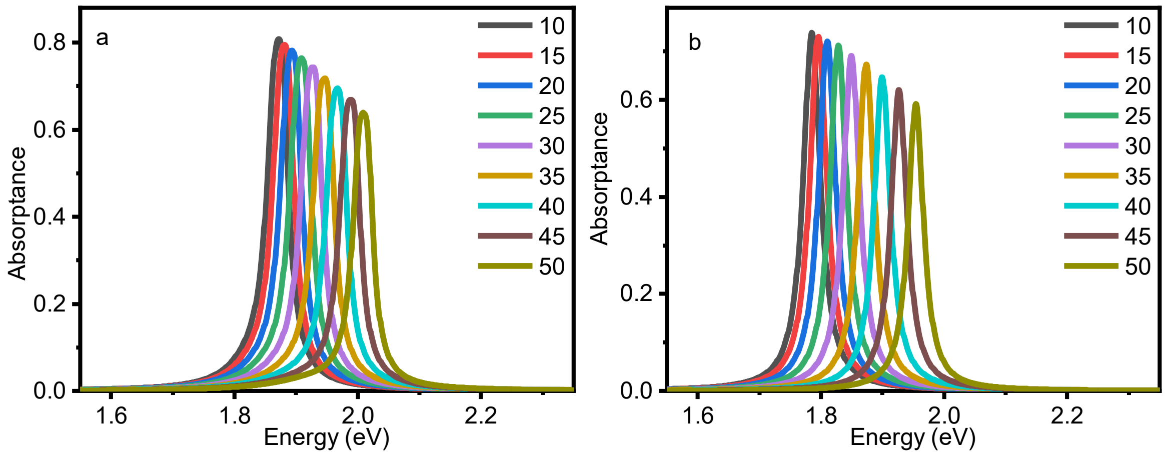

Figure S17. a) The optical visibility of the lower polariton (Abs_{LP}) in a cavity with a thickness of 151 nm. b) The microscopic model version of (a).

2.5. Calculation of the integrated part of $k_{ER\rightarrow LP}$, and $k_{LP\rightarrow ER}$ (including Figures S18-20)

The expressions for the rate constants $k_{ER\rightarrow LP}$, and $k_{LP\rightarrow ER}$ were outlined in Equations 5 and 4. Before conducting the kinetic simulations, the integrated components of both expressions were calculated for all cavities. The calculations shown here are specifically for the cavity with a length of 151 nm. For other cavities, similar calculations were performed in the same manner. The integrated components in $k_{ER\rightarrow LP}$, and $k_{LP\rightarrow ER}$ were calculated as follows:

$$\mathrm{Int\_k_{ER\rightarrow LP}} = \int_0^{\infty} \mathrm{Abs_{LP}}\,(E,\theta) * \mathrm{Em_{film}}(E) * \mathrm{d}E \quad \text{(S11)}$$

$$\mathrm{Int\_k_{LP\rightarrow ER}} = \int_0^{\infty} \mathrm{Abs_{film}}\,(E) * \mathrm{e}^{-\frac{E-\mathrm{E_{LP}}(\theta)}{\mathrm{k_B T}}} * \mathrm{d}E \quad \text{(S12)}$$

$$\mathrm{k_B} = 8.617333262 * 10^{-5}\ \mathrm{eV/K}$$

$$\mathrm{T} = 290\mathrm{K}$$

Here, Abs_{LP}, and Abs_{film} represents the optical visibility of the lower polariton and the area normalized absorption spectrum of the film, respectively (Supplementary section 2.4). Em_{film} refers to the area-normalized spectra of the emission of the film (Figure S18), while E_{LP} denotes the energy of the lower polariton at the reflectance minimum.

The first calculation was done for $Int\_k_{ER\rightarrow LP}$. The spectral multiplication of $Abs_{LP}(E, \theta)$ and $Em_{film}(E)$ was performed (Figures S17-19), and this product was integrated to calculate $Int\_k_{ER\rightarrow LP}$ at each angle.

To calculate $Int\_k_{LP\rightarrow ER}$, the exponential component in $Int\_k_{LP\rightarrow ER}$ was evaluated for all energies and set to 1 for $E < E_{LP}$ (Figure S20). Subsequently, this exponential part was multiplied by $Abs_{film}(E)$, and the resulting product was integrated to calculate $Int\_k_{LP\rightarrow ER}$ at each angle.

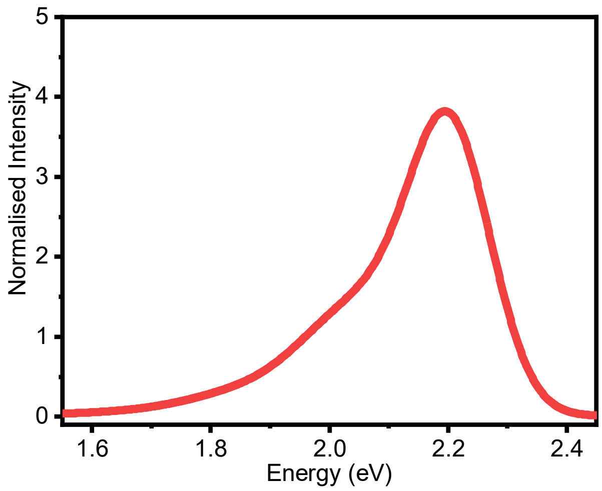

Figure S18. Area-normalized emission spectra of the BODIPY film (Em_{film}).

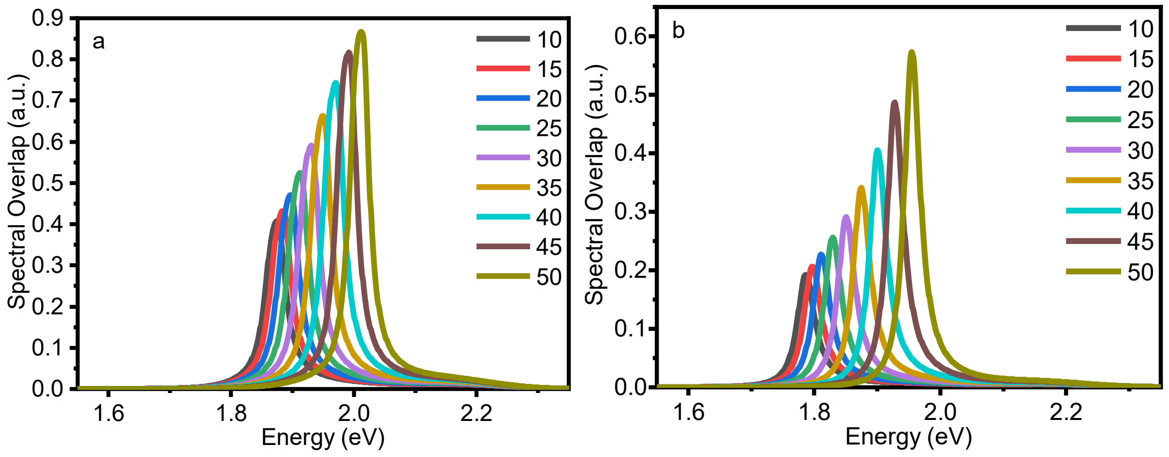

Figure S19. The result of the multiplication of Abs_{LP} and Em_{film} for the cavity with a thickness of 151 nm, calculated using a) experimental values, and b) the microscopic model.

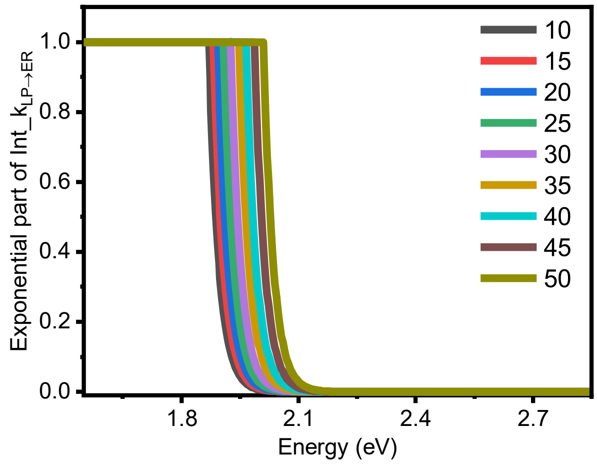

Figure S20. Exponential part of Int_$k_{LP\rightarrow ER}$ for a cavity with a thickness of 151 nm.

2.6. Motivation why the transition into the lower polariton follow the Fermi's golden rule

Assuming Kasha's rule, an excitation resides in the exciton reservoir (ER) as a relaxed excitation on the S_1 potential energy surface of an individual molecule. From this state, radiative decay occurs into the polaritonic modes, which constitute the only optical modes available within the cavity. Within the framework of Fermi's Golden Rule, the rate of emission into the lower polariton (LP) is proportional to the square of the single-molecule light-matter coupling strength, g, and to the photonic Hopfield coefficient squared, $\left|C_{ph}(\theta)\right|^2$.

To rationalize this dependence, we consider a system of N molecules coupled to a single cavity mode. One molecule is detuned and represents the relaxed emitter in the S_1 minimum, while the remaining $N-1$ molecules are resonant with the cavity mode. Within the Tavis-Cummings framework, each molecule couples to the cavity field via the single-molecule coupling strength $g = \boldsymbol{\mu} \cdot \mathbf{f}_c$, where $\boldsymbol{\mu}$ is the transition dipole moment (assumed identical for the BODIPY molecules at ground- and excited-state equilibrium geometries; see Section 2.7), and $\mathbf{f}_c$ is the vacuum field amplitude of the cavity mode. The latter scales with the inverse square root of the mode volume, $\mathbf{f}_c = \sqrt{\hbar\omega_c/(2\epsilon_0\epsilon_r V_{cav})}\,\mathbf{u}_c$, where $\mathbf{u}_c$ is the polarization unit vector. Diagonalization of the Tavis-Cummings Hamiltonian for the N - 1 resonant molecules (Eq. S20) yields N polaritonic eigenstates,

$$|\psi_m\rangle = \left(\sum_j^{N-1} C_{ex,j}^{(\mathrm{m})}\hat{\sigma}_j^+ + C_{ph}^{(m)}\hat{a}^\dagger\right)|\phi_0\rangle \qquad \text{(S13)}$$

where $|\phi_0\rangle$ denotes the global ground state without any excitation (Eq. S23), and $\hat{\sigma}_j^+$ and $\hat{a}^\dagger$ create a molecular excitation and a cavity photon, respectively. The coefficients $C_{ex,j}^{(m)}$ and $C_{ph}^{(m)}$ are the Hopfield amplitudes. The effective coupling between the Nth emitter state $\hat{\sigma}_j^+|\phi_0\rangle$ and a polaritonic eigenstate $|\psi_m\rangle$ is then given by

$$V_{n,m} = \langle\psi_m|\hat{H}^{TC}\hat{\sigma}_N^+|\phi_0\rangle = g_N C_{ph}^{(m)*} \qquad \text{(S14)}$$

showing explicitly that the transition matrix element is proportional to the photonic component of the polariton. Consequently, the emission rate into a given polaritonic mode scales as $|g|^2\left|C_{ph}^{(m)}\right|^2$. The rate further depends on the density of available final states. For the LP branch, we approximate this by a Lorentzian lineshape,

$$\rho_{LP}(E,\theta) = \frac{1}{\pi}\frac{\Gamma_{LP}(\theta)/2}{\left(E-E_{LP}(\theta)\right)^2+(\Gamma_{LP}(\theta)/2)^2} \qquad \text{(S15)}$$

where Γ_{LP} is the LP linewidth. The total emission rate is then obtained by integrating over energy,

$$k_{ER\to LP}(\theta) = \int \frac{2\pi}{\hbar}|g|^2\left|C_{ph}^{LP}(\theta)\right|^2 Em_{film}(E)\,\rho_{LP}(E,\theta)dE \qquad \text{(S16)}$$

where $Em_{film}(E)$ is the normalized molecular emission spectrum. Following Lidzey and co-workers,[6] we define the visibility of the LP as the product of the photonic weight and the density of states,

$$I_{LP}(E,\theta) = \left|C_{ph}^{LP}(\theta)\right|^2\rho_{LP}(E,\theta) \qquad \text{(S17)}$$

Approximating the experimentally accessible absorptance by this optical visibility, $Abs_{LP}(E,\theta) \approx I_{LP}(E,\theta)$, the rate expression can be recast as

$$k_{ER\to LP}(\theta) = \frac{2\pi}{\hbar}|g|^2\int Em_{film}(E)\,Abs_{LP}(E,\theta)dE \qquad \text{(S18)}$$

In this formulation, the Hopfield coefficient is implicitly contained within Abs_{LP}. We emphasize that $Em_{film}(E)$ is normalized, consistent with the single-excitation regime under weak driving conditions, where at most one molecule contributes to emission.

We note that, strictly speaking, the density of states entering Fermi's Golden Rule is not identical to the optical absorptance spectrum, which reflects the linear response of the system to an external electromagnetic field. However, in a cavity system, coupling to external radiation occurs exclusively through the photonic component of the polaritonic states. As a result, the experimentally measured optical visibility already incorporates the photonic Hopfield coefficient, as only this component couples to the external field. Within input-output theory, the absorptance of the lower polariton is therefore proportional to the product of its photonic weight and its spectral density. This motivates the approximation

$$Abs_{LP}(E,\theta) \propto \left|C_{ph}^{(LP)}(\theta)\right|^2 \rho_{LP}(E,\theta) \tag{S19}$$

which avoids double counting of the photonic contribution when evaluating emission rates. Importantly, we find that explicitly including an additional factor of $\left|C_{ph}^{(LP)}(\theta)\right|^2$ in the rate expression leads to a systematic deterioration of the agreement with experiment across all cavities and angles, consistent with the interpretation that this contribution is already contained in the measured absorptance spectra.

2.7. Electronic structure calculations of BODIPY (including Figure S21)

In our model, the rate constant for radiative pumping of the lower polariton from the exciton reservoir is assumed to depend on the single-molecule coupling strength g between the molecular absorption and the cavity electric field (Equation 5 in the main text), which is only valid if the transition dipole moments associated with molecular absorption and emission are similar. To justify this assumption, we performed electronic structure calculations of the BODIPY derivative depicted in Figure S21, which is identical to the molecule used in the experiment, except for the absence of terminal carbon chains, as well as two CN-groups and two carbon atoms linked to the aromatic rings (Figure 1a in the main text).

First, the molecular geometry was optimized in the ground state (S_0) and the first excited state (S_1) with density functional theory[7] (DFT) and time-dependent DFT[8] (TD-DFT) within the Tamm–Dancoff approximation,[9] using the B3LYP[10, 11] exchange-correlation functional and the 6-31G(d) basis set. To implicitly account for solvent effects, the conductor-like polarizable continuum solvent model was applied with parameters characteristic of toluene. After the geometry optimization, a single-point excited state calculation was performed in the two optimized geometries with a larger basis set, namely 6-311+G(2d,p), to ensure a more accurate estimation of the energy of the S_1 state.[12] The resulting energies were 2.54 eV in the geometry optimized ground state and 2.38 eV in the geometry optimized excited state, both within the acceptable error of 0.2 eV for the DFT compared to the experimental absorption and emission maxima. We note that the introduction of the lacking (compared to the experimental molecule) molecular fragments has only a minor effect on the excitation energy, as was confirmed by performing electronic structure calculations with a smaller basis set.

The oscillator strengths of the $S_0 \rightarrow S_1$ transition in the BODIPY geometries optimized in the ground and first excited states, are very similar, namely f_{S0} = 1.002 and f_{S1} = 0.951, which justifies the use of the light-matter coupling strength g in the expression for the radiative pumping rate from the exciton reservoir to the LP branch.

The electronic structure calculations were performed in ORCA 6.0.[13]

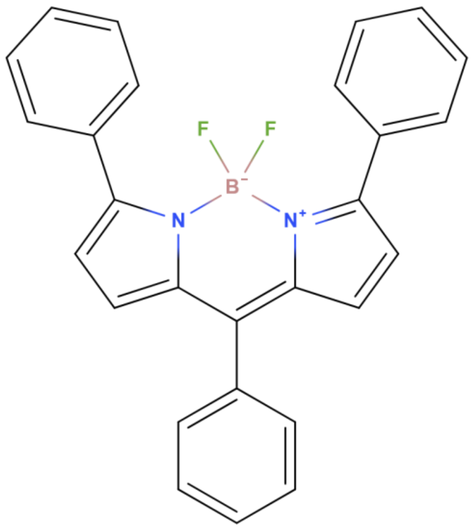

Figure S21. Chemical structure of the BODIPY derivative used in the electronic structure calculations.

2.8. Figure S22

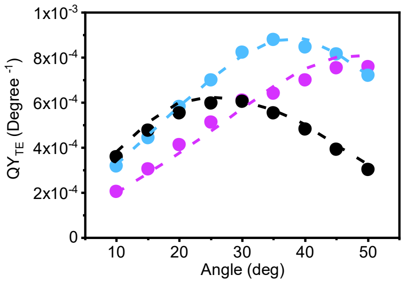

Figure S22. The circles represent the measured emission quantum yields, and the dashed lines represent the quantum yields calculated using the kinetic model. The purple, blue, and black colors correspond to cavities with thicknesses of 141 nm, 133 nm, and 114 nm, respectively.

2.9. Microscopic modeling

The basis of our microscopic model is the Tavis-Cummings Hamiltonian describing the collective coupling of N molecules with excitation energies E_j to a cavity mode with energy E_c:[14]

$$\hat{H}^{\mathrm{TC}} = \sum_j^N E_j\, \hat{\sigma}_j^+ \hat{\sigma}_j^- + E_c \hat{a}_c^\dagger \hat{a}_c + \sum_j^N g_j \left(\hat{\sigma}_j^+ \hat{a}_c + \hat{a}_c^\dagger \hat{\sigma}_j^-\right), \quad \text{(S20)}$$

where the creation and annihilation operators $\hat{a}_c^\dagger$ and $\hat{a}_c$ create and destroy an excitation in the cavity mode, respectively; $\hat{\sigma}_j^+$ and $\hat{\sigma}_j^-$ excite and de-excite molecule j, and g_j is the strength with which molecule j couples to the cavity. The strength is proportional to the inner product between the molecular transition dipole moment, $\boldsymbol{\mu}_j$, and the cavity vacuum field, $\mathbf{u}_c\sqrt{E_c/2\epsilon V_c}$, with unit vector $\mathbf{u}_c$ the field's direction, and V_c the mode volume.

As the basis for this Hamiltonian, we choose product state wave functions, defined as

$$\phi_n = \hat{\sigma}_n^+ |\phi_0\rangle \quad \text{(S21)}$$

for $1 \leq n \leq N$ and

$$\phi_n = \hat{a}_n^\dagger |\phi_0\rangle \quad \text{(S22)}$$

for $n > N$, where

$$\phi_0 = |S_0^1\rangle \otimes |S_0^2\rangle \otimes |S_0^3\rangle \ldots \otimes |S_0^N\rangle \otimes |0_c\rangle \quad \text{(S23)}$$

is the product state where neither the molecules nor the cavity mode is excited. The molecules are thus in their electronic ground states $\left(S_0^j\right)$ and cavity mode is empty (0).

For the ideal situation, in which all molecules can be described as identical two-level systems, diagonalization of the Hamiltonian yields two bright polaritonic eigenstates, characterized by a contribution from the cavity mode excitation, and N-1 dark states that lack such contribution. In what follows, we use this ideal situation as a reference for understanding the effects of disorder, which causes each of the molecules to have different excitation energies. For simplicity, we keep assuming that the single-molecule coupling strength, $g = g_0/\sqrt{L}$, remains the same for all molecules.

We model the molecule-cavity system with N = 2000 molecules. To account for their disorder, we deterministically sampled their energies from the BODIPY absorption spectra, calculated with TMM (Figure S15) from 1.0 to 3.0 eV. As in the experiments, we consider n_w = 7 cavity widths, w_m, and n_θ = 9 angles, θ_k. The energies of the cavity modes, $E_c(w_m, \theta_k)$, were extracted from TMM calculations on an empty cavity system, in which the molecular layer was modeled by the static real part of the BODIPY dielectric function, $\epsilon_\infty = 2.34$. The cavity mode decay rates, γ_c, were obtained by fitting Lorentzian functions to the absorption spectrum of the empty cavity. These losses were added to the cavity energy term in Equation S20: $E_c - \frac{i}{2}\hbar\gamma_c$. For the molecules a dephasing rate of 10 meV was assumed. The single-molecule coupling strength parameter,[15] g_0, was set to 8.7 meV, which yielded slightly smaller Rabi splittings than in experiment, but avoids that the upper polariton extends into the high-energy region where the refractive index of molecular absorption becomes inaccurate.

For $n_w \times n_\theta$ cavity model systems, we diagonalized the Tavis-Cummings Hamiltonian to obtain the N + 1 complex eigenenergies. Spectra were created as sum of Lorentzians, centered at the real parts of these energies, and their imaginary parts as the width parameters. In Figure S24, we plot the angle-dependent Rabi splittings as a function of cavity length.

Overlaps were computed by first fitting a Lorentzian to the lower polariton and numerically integrating the product of that fit with the absorption or emission spectra. For the 151 nm cavity, the overlap between the molecular and polariton absorption is plotted in Figure S23 and the overlap between the LP absorption and the BODIPY emission is plotted in Figure S19.

Because the inverse participation ratio (IPR, equation 8 in the main text) requires the expansion (Hopfield) coefficients, we also diagonalized the Hamiltonian without the loss and dephasing terms. The eigenstates with the highest photonic content were identified as the polaritonic states and their IPR was computed.

2.10. An approximation of the number of molecules in the mode volume

An approximate number of molecules in the mode volume was calculated as follows. Assuming a density of the molecular film of 1 g/cm^3 and a molecular weight of 694.72 g/mol results in 8.7x10^{20} molecules per cubic centimeter. Assuming a diffraction limited mode volume in a lambda half cavity, the mode volume should be on the order of the cube of half the excitation wavelength. The cube of 500/2 nm is 1.6 x10^{-14} cm^3. Thus, a rough number of the number of molecules in the mode volume would be 8.7x10^{20} x 1.6 x10^{-14} = 1.4 x10^7, which is in the same order of magnitude as our calculated value.

Alternatively, we can compute the mode volume, V_c, directly from the single-molecule coupling strength, which depends on the mode volume via

$$g = \frac{1}{3}|\mu|\sqrt{E_m/(2\epsilon_0\epsilon_r V_c)}$$

With μ the BODIPY transition dipole moment, E_m the molecular excitation energy, and ϵ_0 and ϵ_r the vacuum permittivity and relative permittivity, respectively. The factor $\frac{1}{3}$ accounts for the random orientations of the molecules in the film.

Using the dipole moment from the TDDFT modeling (μ = 10.2 Debeye, see Section 2.7), the relative permittivity from the TMM calculations (ϵ_r = 2.34), and an average excitation energy of E_m = 2.41 eV, we find that the mode volume scales linearly with the cavity length, L, as: $V_c = 6.96\ 10^4 L$.

Assuming a density of 1.0 g/cm^3, we obtain estimates for the number of molecules, *N,* listed in Table S3, that are in good agreement with the number of molecules determined from the collective coupling strengths (Equation 7, main text).

2.11. Calculation of the overlap between the absorption of the LP and the film (including Figure S23)

To calculate the overlap, spectral multiplication between the area normalized Abs_{LP} (Figure S23a) and Abs_{film} (Figure S23b) was first conducted (Figure S23c). The overlap integral was then determined by integration.

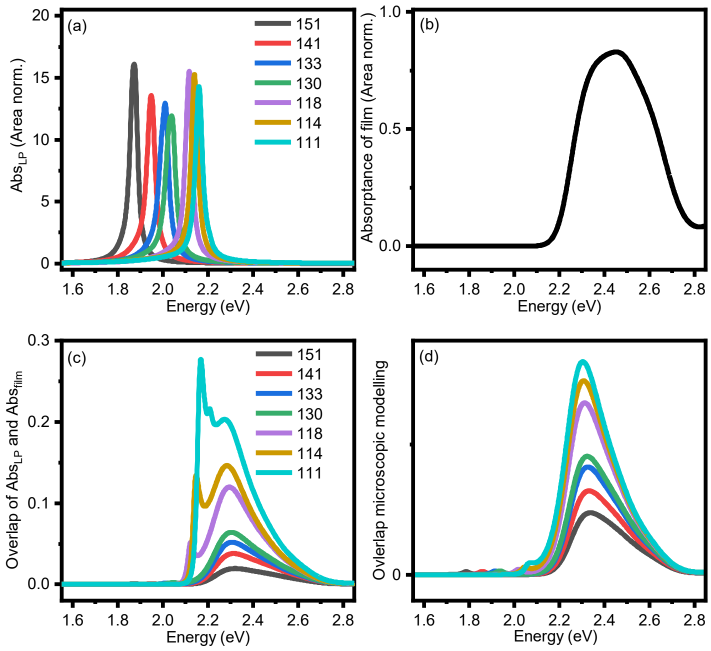

Figure S23. a) Area normalized Abs_{LP} at 10 degrees of the different cavities. b) Replot of the TMM calculated Abs_{film} for a 151 nm thick BODIY film (same as Figure S15). c) Multiplication of the area normalized Abs_{LP} and Abs_{film}. d) The same calculated using the microscopic model.

2.12. Figure S24

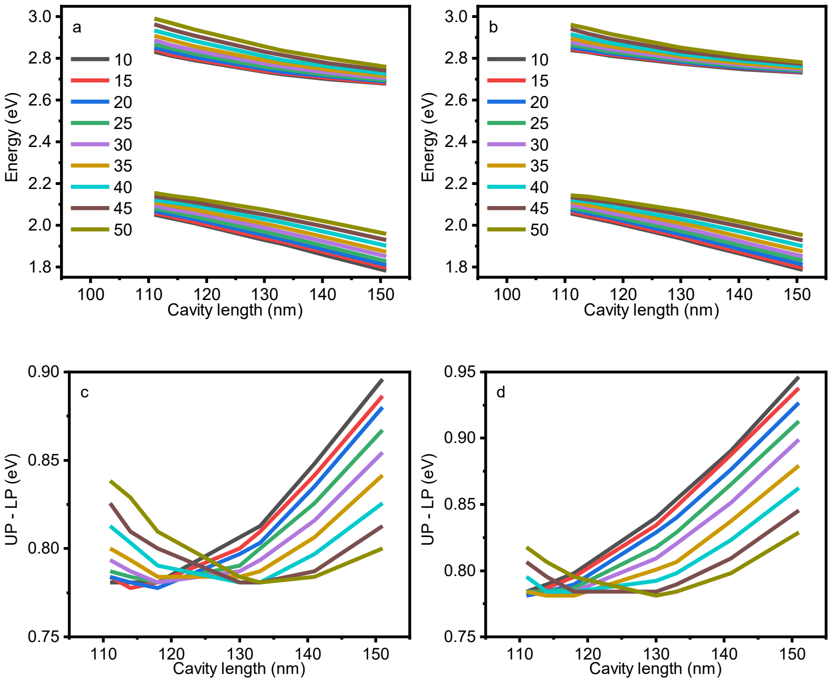

Figure S24. Energy of the Upper and Lower polariton a) for an ideal system, and b) taking into account disorder in the calculations. Energy difference between UP and LP for the different cavity lengths and angels c) for an ideal system, and d) taking into account disorder.

3. Supplementary Tables

Table S1. The parameters were extracted from the TMM and the CHO model. Where L_{active} is the thicknesses of the molecular layer inside the cavity, extracted from the TMM fitting. g_1, g_2 , and g_N are the coupling strengths between E_{X1} (2.379 eV) and the cavity mode, and E_{X2} (2.543 eV) and cavity mode, and total coupling strength respectively. $E_C(0)$ and $Hop_{Ph}(0)$ are the cavity energy and the photonic fraction of LP at $k_{||}$ =0, respectively.

$L_{acvtive}$ (nm)	g_1 (meV)	g_2 (meV)	g_N (meV)	$E_C(0)$ (eV)	$Hop_{Ph}(0)$
151	171.3	225.5	396.8	1.98718	0.8227
141	178	226.7	404.7	2.08754	0.7713
133	185.6	221.9	407.5	2.17163	0.7176
130	192.3	219.4	411.7	2.21825	0.6815
118	208.7	206.2	414.9	2.35741	0.5587
114	218.4	195.5	413.9	2.41245	0.5037
111	226.3	184.3	410.6	2.46032	0.4555

Table S2. The parameters extracted from the LP emission QY modeling using the rate law equations. Where QY is the total emission quantum yield from the cavity measured using an integrated sphere. The cavity thickness normalized single molecular coupling strength (g_0) and C_3 are the two fitting parameters in the modeling.

L_{cavity} (nm)	QY_{exp}	g_0	g (meV)	C_3 (10^{14})
151	0.0334	0.00142	0.1154	9.638
141	0.0426	0.00142	0.1195	9.638
133	0.0502	0.00142	0.123	9.638
130	0.0497	0.00142	0.1244	9.638
118	0.0396	0.00142	0.1306	9.638
114	0.0322	0.00142	0.1329	9.638
111	0.0276	0.00142	0.1346	9.638

Table S3. Mode volumes, V_c, and number of molecules, N, extracted from the single-molecule coupling strengths, under the assumption of 1.0 gcm^{-3} density (Section 2.10).

L_{cavity} (nm)	V_c (10^7 nm^3)	N (10^6)
151	10.5	9.15
141	9.80	8.53
133	9.25	8.05
130	9.05	7.87
118	8.21	7.14
114	7.93	6.90
111	7.73	6.72

4. References

1. Bhuyan, R.; Lednev, M.; Schäfer, C.; Feist, J.; Börjesson, K. Quantitative Modeling of Polaritonic Emission Using the Source Term Method. *J Phys Chem Lett* **2025**, *16* (25), 6435-6441.
2. Lakowicz, J. R. Principles of Fluorescence Spectroscopy. *Boston, MA: springer US* **2006**.
3. Godsi, M.; Golombek, A.; Balasubrahmaniyam, M.; Schwartz, T. Exploring the nature of high-order cavity polaritons under the coupling-decoupling transition. *J Chem Phys* **2023**, *159* (13), 134307.
4. Byrnes, S. J. Multilayer optical calculations. *arXiv* **2020**, 1603.02720.
5. M.J., W. Handbook of Optical Materials. *CRC Press* **2002**.
6. Lidzey, D. G.; Bradley, D. D. C.; Skolnick, M. S.; Virgili, T.; Walker, S.; Whittaker, D. M. Strong exciton-photon coupling in an organic semiconductor microcavity. *Nature* **1998**, *395* (6697), 53-55.
7. Hohenberg, P.; Kohn, W. Inhomogeneous electron gas. *Phys Rev* **1964**, *136* (3B), B864.
8. Runge, E.; Gross, E. K. Density-functional theory for time-dependent systems. *Phys Rev Lett* **1984**, *52* (12), 997.
9. Hirata, S.; Head-Gordon, M. Time-dependent density functional theory within the Tamm–Dancoff approximation. *Chemical Physics Letters* **1999**, *314* (3-4), 291-299.
10. Becke, A. D. Density-functional thermochemistry. III. The role of exact exchange. *J Chem Phys* **1993**, *98* (7), 5648-5652.
11. Stephens, P. J.; Devlin, F. J.; Chabalowski, C. F.; Frisch, M. J. Ab initio calculation of vibrational absorption and circular dichroism spectra using density functional force fields. *The Journal of physical chemistry* **1994**, *98* (45), 11623-11627.
12. Le Guennic, B.; Jacquemin, D. Taking up the cyanine challenge with quantum tools. *Accounts Chem Res* **2015**, *48* (3), 530-537.
13. Neese, F. The ORCA program system, WIREs Comput. Mol. Sci. 2 (2012) 73–78.
14. Tavis, M.; Cummings, F. W. Approximate Solutions for an N-Molecule-Radiation-Field Hamiltonian. *Phys Rev* **1969**, *188* (2), 692-695.
15. Sokolovskii, I.; Morozov, D.; Groenhof, G. One molecule to couple them all: Toward realistic numbers of molecules in multiscale molecular dynamics simulations of exciton-polaritons. *J Chem Phys* **2024**, *161* (13), 134106.
